# The Breadth and Molecular Basis of Hcp-Driven Type VI Secretion System Effector Delivery

**DOI:** 10.1128/mBio.00262-21

**Published:** 2021-06-01

**Authors:** Sophie A. Howard, R. Christopher D. Furniss, Dora Bonini, Himani Amin, Patricia Paracuellos, David Zlotkin, Tiago R. D. Costa, Asaf Levy, Despoina A. I. Mavridou, Alain Filloux

**Affiliations:** a Imperial College London, Department of Life Sciences, MRC Centre for Molecular Bacteriology and Infection, London, United Kingdom; b Science for Life Laboratory, Department of Molecular Biosciences, The Wenner-Gren Institute, Stockholm University, Stockholm, Sweden; c Department of Molecular Genetics, Weizmann Institute of Science, Rehovot, Israel; d Department of Molecular Biosciences, University of Texas at Austin, Austin, Texas, USA; Nanyang Technological University

**Keywords:** T6SS, Hcp, *Pseudomonas*, protein secretion, toxin

## Abstract

The type VI secretion system (T6SS) is a bacterial nanoscale weapon that delivers toxins into prey ranging from bacteria and fungi to animal hosts. The cytosolic contractile sheath of the system wraps around stacked hexameric rings of Hcp proteins, which form an inner tube. At the tip of this tube is a puncturing device comprising a trimeric VgrG topped by a monomeric PAAR protein. The number of toxins a single system delivers per firing event remains unknown, since effectors can be loaded on diverse sites of the T6SS apparatus, notably the inner tube and the puncturing device. Each VgrG or PAAR can bind one effector, and additional effector cargoes can be carried in the Hcp ring lumen. While many VgrG- and PAAR-bound toxins have been characterized, to date, very few Hcp-bound effectors are known. Here, we used 3 known Pseudomonas aeruginosa Hcp proteins (Hcp1 to -3), each of which associates with one of the three T6SSs in this organism (H1-T6SS, H2-T6SS, and H3-T6SS), to perform *in vivo* pulldown assays. We confirmed the known interactions of Hcp1 with Tse1 to -4, further copurified a Hcp1-Tse4 complex, and identified potential novel Hcp1-bound effectors. Moreover, we demonstrated that Hcp2 and Hcp3 can shuttle T6SS cargoes toxic to Escherichia coli. Finally, we used a Tse1-Bla chimera to probe the loading strategy for Hcp passengers and found that while large effectors can be loaded onto Hcp, the formed complex jams the system, abrogating T6SS function.

## INTRODUCTION

Bacterial secretion systems shuttle proteins to the extracellular milieu or into target cells (1, 2). The type VI secretion system (T6SS) functions as a contractile device that resembles a bacteriophage (3, 4). The contractile sheath assembles onto a baseplate (TssKEFG) which is bound to the cytoplasmic membrane via an interaction with the membrane complex TssLMJ. The sheath made of TssBC protomers (5–8) extends in the cytosol while wrapping around a stack of hexameric Hcp rings (9) until it reaches the opposite side of the cell (10). The Hcp rings fit within the sheath lumen and pile on top of each other (11, 12). The formation of the Hcp tube is dependent on a spike structure, the VgrG trimer, whose large base provides the interface for assembly of the first Hcp ring (13). At the other end of VgrG sits a conical PAAR monomer (14). The PAAR-VgrG-Hcp could be compared to a spear, with the projectile point being the VgrG-PAAR complex and the shaft the Hcp tube. Sheath contraction results in spear ejection and, cryotomograms of the contracted sheath show hollow structures (10, 15).

The T6SS has been shown to be important in bacterium-host interaction but is also an antimicrobial weapon that injects toxins into bacterial or fungal prey cells (16, 17). The T6SS effectors have no recognizable signals, except on some occasions where they are found to contain MIX or FIX motifs (18, 19). These effectors are loaded on the PAAR, VgrG, or Hcp components of the T6SS apparatus. This can occur either through direct protein-protein interactions (20) or with the help of chaperones/adaptors such as EagR (21–23) or Tap proteins (24, 25). Effector proteins fused to PAAR, VgrG, or Hcp protein have also been identified and are termed specialized effectors (26–30) as opposed to cargo effectors associated with the T6SS via protein-protein interactions.

A large number of VgrG- and PAAR-associated effectors, either specialized or cargo, have been functionally characterized (28, 29, 31, 32). For Hcp, few specialized effectors have been reported for the Escherichia coli T6SS (33), while the Pseudomonas aeruginosa Tse2 cargo is loaded within the Hcp1 ring lumen (34). Hcp1 is not the only Hcp protein found in P. aeruginosa, and at least two additional Hcp’s, Hcp2 and Hcp3, could carry effector cargo. In this study, we used all three P. aeruginosa Hcp proteins to perform an *in vivo* pulldown approach. We confirmed that Hcp1 binds to Tse1 to -4, as reported previously (34, 35), and identified novel Hcp1-bound proteins, some in the size range of 10 to 30 kDa that might be suited to fit into the Hcp lumen. We were also able to identify Hcp2- and Hcp3-bound proteins, some of which exert toxicity when produced in the cytosol of Escherichia coli. Moreover, we found that conserved Hcp1 to -3 inner ring residues were required for successful interaction with both the known and the uncharacterized effectors. Moreover, we investigated the size limit for Hcp cargoes by using chimeric proteins. We observed that large chimeric effectors bound to Hcp can be recognized by the T6SS but jam the system, likely because of steric hindrance during the assembly of the T6SS sheath and tube. We conclude that Hcp-dependent transport is a widely used strategy for the delivery of T6SS cargo effectors but has limitations compared to VgrG-dependent transport, which allows for translocation of larger effectors.

## RESULTS

### Biochemical characterization of Tse1 to -4/Hcp interactions.

Tse1 to -3 were the first T6SS antibacterial toxins identified (36) through a secretome analysis. It was later shown that the presence of Hcp1 can stabilize these effectors and proposed that Tse2 fills the lumen of the Hcp ring (34). Here, we produced a His-tagged version of Hcp1 (see Fig. S1 in the supplemental material) and hemagglutinin (HA)-tagged versions of the catalytically inactive Tse1 to -3 toxins, i.e., Tse1C30A, Tse2T79A/S80A, and Tse3E250Q (37, 38). We demonstrate that interaction could indeed be identified using our pulldown approach where the *hcp1*-encoding plasmid and one of the plasmid-encoding toxins were cotransformed in E. coli. Upon copurification using Hcp1 as a bait, elution from Ni-nitrilotriacetic acid (NTA) resin and size exclusion chromatography, Tse1, Tse2, and Tse3 were found in complex with Hcp1 (see Fig. S2).

10.1128/mBio.00262-21.1 FIG S1 Hcp1 ring structure. (A) Coomassie blue stain of Hcp1-His purified via Ni resin and then run through a SD200 SEC column for further clarification. Lanes labeled on top are postexpression sample, insoluble and soluble samples after sonication and clarification, flowthrough (FT) from the Ni-NTA column, wash fraction before elution, Ni fractions corresponding to the elution peak, and SEC fractions corresponding to the gel filtration peak. Molecular weight standards are on the left. (B) Representative negative-stain electron micrograph of Hcp1 with 2D class averages showing top and side view orientations. (C) SEC chromatograph of purified Hcp1-His. Download FIG S1, TIF file, 2.0 MB. Copyright © 2021 Howard et al. 2021 Howard et al. https://creativecommons.org/licenses/by/4.0/ This content is distributed under the terms of the Creative Commons Attribution 4.0 International license.

10.1128/mBio.00262-21.2 FIG S2 Confirmation of Hcp1 interaction with Tse1 to -3. (A) Western blot of Hcp1-His and Tse1^C30A^-HA copurified via Ni resin and a SD200 SEC column; lanes labeled on the top are postexpression sample, insoluble and soluble samples after sonication and clarification, flowthrough (FT) from the Ni-NTA column, wash fraction before elution, Ni fractions corresponding to the elution peak, and SEC fractions corresponding to the gel filtration peak. The antibodies used are labeled on the right: top panels anti-HA antibody (BioLegend) at 1:1,000 concentration; bottom, anti-His antibody (GenScript) at 1:1,000 concentration. Molecular weight standards are on the left. (B) SEC chromatograph of gel-filtrated Hcp1-His and Tse1^C30A^-HA copurification. (C) Western blot of Tse1^C30A^-HA alone purification for negative control. (D) Western blot of Hcp1-His and HA-Tse2^T79A S80A^ copurification. (E) SEC chromatograph of gel filtrated Hcp1-His and HA-Tse2^T79A S80A^ copurification. (F) Western blot of HA-Tse2^T79A S80A^ alone purification for negative control. (G) Western blot of Hcp1-His and Tse3^E250Q^-HA copurification. (H) SEC chromatograph of gel-filtrated Hcp1-His and Tse3^E250Q^-HA copurification. (I) Western blot of Tse3^E250Q^-HA alone purification for negative control. Download FIG S2, TIFF file, 1.3 MB. Copyright © 2021 Howard et al. 2021 Howard et al. https://creativecommons.org/licenses/by/4.0/ This content is distributed under the terms of the Creative Commons Attribution 4.0 International license.

**FIG 1:**
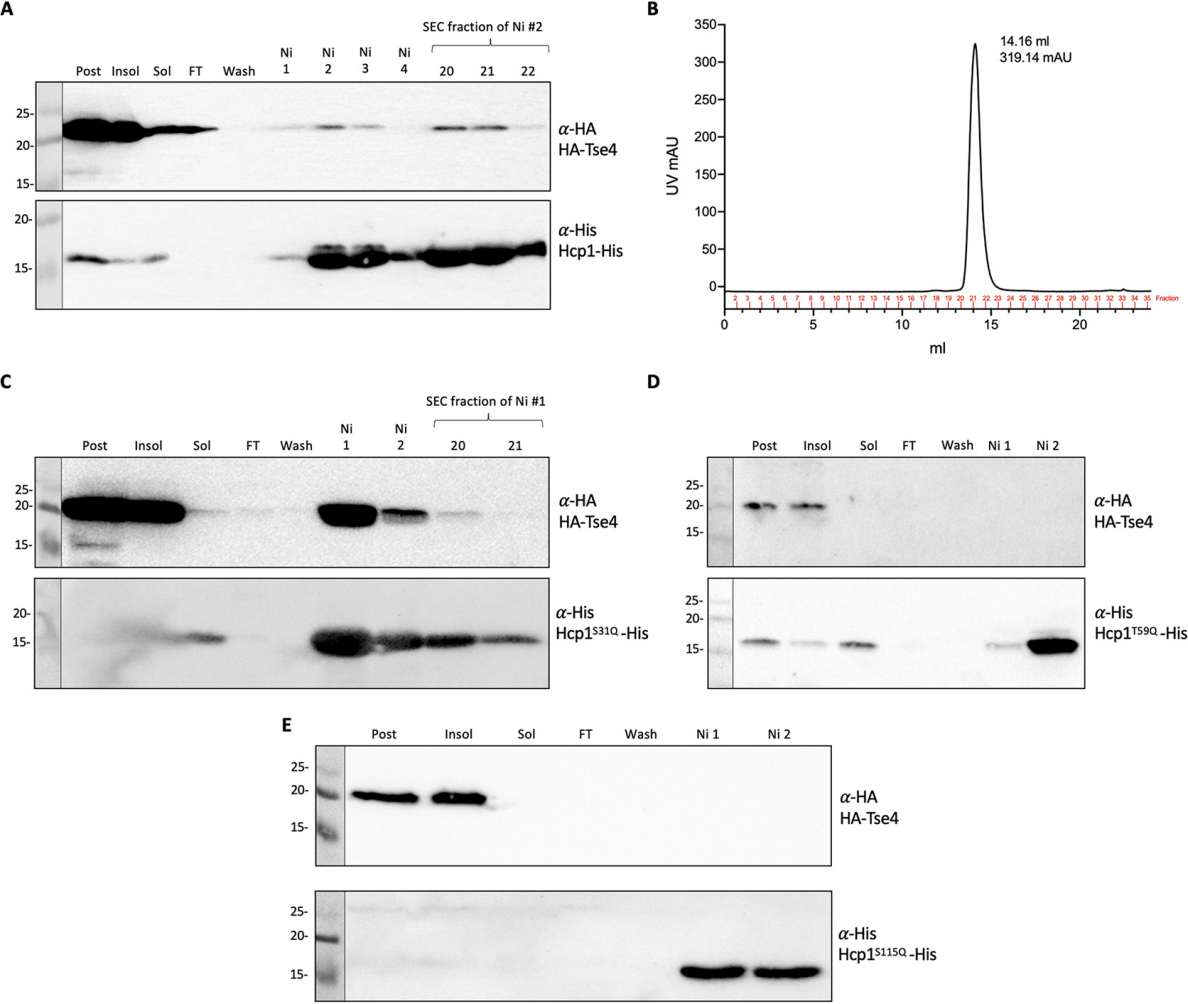
HA-Tse4 interacts specifically with Hcp1 inner ring residues. (A) Western blot of Hcp1-His and HA-Tse4 copurified via Ni resin and an SD200 SEC column; lanes labeled on the top are postexpression sample, insoluble and soluble samples after sonication and clarification, flowthrough (FT) from the Ni-NTA column, wash fraction before elution, Ni fractions corresponding to the elution peak, and SEC fractions corresponding to the gel filtration peak. The antibodies used are labeled on the right of the Western blot; top, anti-HA (BioLegend) at 1:1,000 concentration; bottom, anti-His (GenScript) at 1:1,000 concentration. Molecular weight standards are on the left. (B) SEC chromatograph of gel-filtrated Hcp1-His and HA-Tse4 copurification. Western blots of Hcp1-His inner ring mutants and HA-Tse4 copurifications with Hcp1^S31Q^-His (C), with Hcp1^T59Q^-His (D), and with Hcp1^S115Q^-His (E).

The P. aeruginosa Tse4 toxin was discovered using quantitative cellular proteomics by comparing wild-type and *hcp1* mutant strains and observing a low abundance of Tse4 in an *hcp1* mutant background (35). Tse4 is a pore-forming toxin that allows small ion leakage (39). We assessed a direct interaction between Hcp1 and Tse4 by using the pulldown approach. Overexpression of Tse4 in E. coli is toxic. We observed that adding a C-terminal HA tag, rather than an N-terminal tag, decreased the level of toxicity (see Fig. S3A). However, the solubility of Tse4-HA was limited, while copurification with Hcp1-His showed almost no interaction (Fig. S3B and C). We hypothesized that the C-terminal HA tag interferes with Tse4 activity but also impairs interaction with Hcp1. Instead, while an N-terminally tagged HA-Tse4 was not soluble, its solubility was improved upon coexpression with Hcp1-His, and copurification resulted in a Tse4-Hcp1 complex that was maintained after size exclusion chromatography (Fig. 1A and B).

10.1128/mBio.00262-21.3 FIG S3 Solubility, toxicity, and purification of Tse4. E. coli BL21 cells expressed either HA-tagged Tse4 from pET22b alone or with Hcp1-His from pACYCDuet-1. (A) Toxicity assay with cells plated on uninduced (1% glucose) or induced (50 μM IPTG) agar for 16 h at 37°C. Strains are labeled on the *x* axis. Means and SEMs are from three biological replicates. An unpaired *t* test was used between the uninduced and induced samples to determine statistical significance. *, *P* < 0.05. (B) After induction, cells were grown at 18°C overnight, harvested, and resuspended in purification resuspension buffer, sonicated, and centrifuged. Western blot of postexpression, insoluble and soluble samples; lanes labeled on top are the proteins expressed in BL21. Antibodies used are labeled on the right: top, anti-HA antibody (BioLegend) 1:1000; bottom, anti-His antibody (GenScript) 1:1,000. Molecular weight standards are on the left. (C) Western blot of Hcp1-His and Tse4-HA copurified via Ni resin and a SD200 SEC column; lanes labeled on the top are postexpression sample, insoluble and soluble samples after sonication and clarification, flowthrough (FT) from the Ni-NTA column, wash fraction before elution, and Ni fractions corresponding to the elution peak. Download FIG S3, TIFF file, 0.9 MB. Copyright © 2021 Howard et al. 2021 Howard et al. https://creativecommons.org/licenses/by/4.0/ This content is distributed under the terms of the Creative Commons Attribution 4.0 International license.

Previous studies identified key residues in the Hcp1 inner ring surface that are important for secretion of Tse1 to -3, namely, S31, which is only important for secretion of Tse2, L28 and A29, which are important for secretion of Tse2 and Tse3, and T59, S115, and T122, which are important for secretion of Tse1, Tse2, and Tse3 (34). We confirmed that S31 is important for interaction with Tse2, while S115 is key for interaction with all 3 toxins, Tse1 to -3 (see Fig. S4). We tested whether these amino acids are also important for the Hcp1-Tse4 interaction and found that the Hcp1S31Q variant is still able to interact with Tse4, although only weakly since the complex is lost after gel filtration, whereas this interaction is totally abrogated when the Hcp1T59Q and Hcp1S115Q variants are used (Fig. 1C to E). We therefore conclude that S31 appears to be specific for Tse2 interaction, while T59 and S115 have a more general role, and their modification disrupts interaction with T6SS toxins.

10.1128/mBio.00262-21.4 FIG S4 Hcp1 inner ring mutants stop interaction with Tse1 to -3. Western blots of Hcp1 inner ring mutants copurified with Tse1 to -3 via Ni resin and a SD200 SEC column; lanes labeled on the top are postexpression sample, insoluble and soluble samples after sonication and clarification, flowthrough (FT) from the Ni-NTA column, wash fraction before elution, Ni fractions corresponding to the elution peak, and SEC fractions corresponding to the gel filtration peak. The antibodies used are labeled on the right: top, anti-HA antibody (BioLegend) at 1:1,000 concentration; bottom, anti-His antibody (GenScript) at 1:1,000 concentration. Molecular weight standards are on the left. (A) Hcp1^S115Q^-His and Tse1^C30A^-HA copurified. (B) Hcp1^S115Q^-His and Tse3^E250Q^-HA copurified. (C) Hcp1^S31Q^-His and HA-Tse2^T79A S80A^ copurified. (D) Hcp1^S115Q^-His and HA-Tse2^T79A S80A^ copurified. Download FIG S4, TIFF file, 1.1 MB. Copyright © 2021 Howard et al. 2021 Howard et al. https://creativecommons.org/licenses/by/4.0/ This content is distributed under the terms of the Creative Commons Attribution 4.0 International license.

### Identification of novel T6SS effectors by *in vivo* Hcp cross-linking.

**FIG 2:**
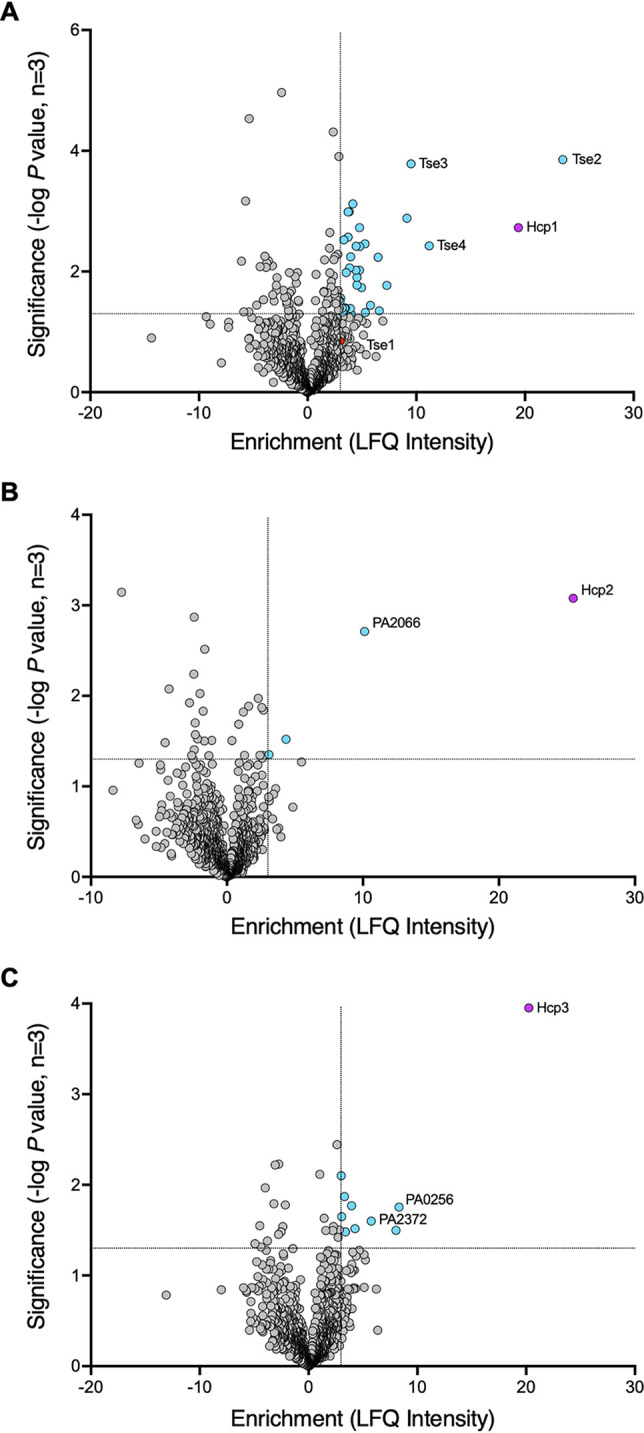
Hcp1/2/3 pulldown hits. Volcano plots showing LFQ analysis of Hcp1-, Hcp2-, and Hcp3-FLAG pulldowns performed in P. aeruginosa. Results are from three independent experiments, and the full data sets are provided in Data Set S1 in the supplemental material. The *x* axes show the fold change in enrichment over the untagged Hcp control. The *y* axes depict the significance of the enrichment. Proteins plotted were not found to be enriched in the cognate VgrG-FLAG controls (Data Set S1). (A) Hcp1-FLAG pulldown results. The red dot is Tse1, a known Hcp1-delivered effector that was enriched 3-fold, but was below the significance cutoff. This was used as the enrichment baseline for possible hits. (B) Hcp2-FLAG pulldown results. (C) Hcp3-FLAG pulldown results. Dotted lines represent the enrichment cutoff of ≥3-fold, as determined by Tse1 and the significance cutoff −log_10_ *P* value of ≥1.3, which is equivalent to a *P* value of ≤0.05. Increasing significance on the *y* axis corresponds to a smaller *P* value. Blue dots are proteins above the enrichment and significance cutoffs. Purple dots are the bait Hcp proteins.

Having validated our pulldown approach using Hcp-His as a reliable bait, we used Hcp1 to -3 bearing a C-terminal FLAG tag for *in vivo* pulldown in P. aeruginosa PAO1 Δ*rsmA*. The rationale for using this strain is that an *rsmA* mutation increases expression of all three T6SSs in P. aeruginosa (40). Lysates of cells were treated with a dimethyl 3,3′-dithiobispropionimidate (DTBP) cross-linker in order to capture transient or weak interactions between Hcp1 to -3 and new putative cargo proteins and were then applied to FLAG M2 magnetic beads; proteins that coeluted with each Hcp were identified by mass spectrometry. For each Hcp-FLAG pulldown, the identified proteins were cross-referenced against the results of two control pulldown experiments: one performed using untagged Hcp as the bait protein and the other using a FLAG-tagged version of each Hcp’s cognate VgrG protein (see Materials and Methods). Proteins found to be significantly enriched (≥3-fold; the level of enrichment of the known Hcp1 effector Tse1) over the untagged control, while at the same time not found to be enriched in the VgrG control pulldown, were considered putative Hcp-bound effectors. In each of the volcano plots depicting the results (Fig. 2), the tagged Hcp protein is, as expected, highly enriched. Initial inspection of the proteins identified in the Hcp pulldowns (Table 1) revealed 107 proteins enriched ≥3-fold by Hcp1, 32 of which are significant Hcp1-specific interacting partners. Of these proteins, 23 (71.88%) have an *M*_w_ equal to or below that of Tse3, which at 44.4 kDa is the largest of the known Hcp1-delivered effectors. Tse2, Tse3, and Tse4 appear as the top 3 hits, followed by the remaining Hcp-interacting proteins, some of which appear to be good candidates for novel Hcp1-bound effector proteins (such as PA3440, an 11.6-kDa protein of unknown function). Applying these same criteria to the Hcp2 and Hcp3 pulldowns revealed 30 proteins enriched ≥3-fold by Hcp2, 2 of which are significant Hcp2-specific interacting partners with molecular weights below that of Tse3 (PA2066 and PA2966) and 63 proteins enriched ≥3-fold by Hcp3, 9 of which are significant Hcp3-specific interacting partners with molecular weights below that of Tse3 (PA0256, PA3187, PA2372 [a 21-kDa protein of unknown function encoded within the H3-T6SS cluster], PA3076, PA0381, PA1373, PA3182, PA4920, and PA0771) (Tables 2 and 3). Finally, in order to ensure that our approach offers an unbiased method for identifying new Hcp-bound T6SS effectors, we compared the theoretical isoelectric point (pI) of the putative Hcp-bound effectors identified in our pulldown experiments to the theoretical average pI of the P. aeruginosa PAO1 proteome. The average pI of the Hcp-bound putative effectors identified here was 6.16 ± 1.29 (range, 3.79 to 9.92), while the average pI of the P. aeruginosa proteome is 7.12 ± 1.96 (range, 3.12 to 13.51). Thus, this approach has no obvious bias for proteins of a certain charge and is able to identify proteins from throughout the P. aeruginosa proteome.

**TABLE 1:** Hcp1-FLAG top interaction hits^*a*^

Protein	Fold change	Significance^*b*^	Description of protein	Size (kDa)
PA2702 Tse2	23.4	3.85	Known Hcp1 effector	17.7
PA2774 Tse4	11.2	2.42	Known Hcp1 effector	19.2
PA3484 Tse3	9.6	3.78	Known Hcp1 effector	44.4
PA2396 PvdF	9.2	2.88	Pyoverdine synthetase F	31
PA2000 DhcB	7.2	1.77	Dehydrocarnitine CoA^*c*^ transferase	23.2
PA0662 ArgC	6.6	1.35	*N*-Acetyl-gamma-glutamyl-phosphate reductase	36.7
PA4907 YdfG	6.4	2.24	Short-chain dehydrogenase	27.4
PA0075 PppA	5.8	1.44	Represses H1-T6SS	25.9
PA5220	5.3	1.32	Hypothetical	30.5
PA4748 TpiA	5.3	2.46	Triosephosphate isomerase	25.6
PA0895 AruC	5	1.73	Succinylornithine transaminase/acetylornithine aminotransferase	43.7
PA3471 MaeA	4.8	2.41	NAD-dependent malic enzyme	62.4
PA1588 SucC	4.8	2.73	Succinate-CoA ligase (ADP-forming) subunit beta	41.5
PA0317	4.8	2.02	d-2-Hydroxyglutarate dehydrogenase	51.3
PA1589 SucD	4.5	1.78	Succinate-CoA ligase (ADP-forming) subunit alpha	30.3
PA5427 AdhA	4.5	1.90	Alcohol dehydrogenase	35.9
PA2553	4.5	2.42	Probable acyl-CoA thiolase	41.4
PA4854 PurH	4.4	2.02	Bifunctional purine biosynthesis protein	57.7
PA2623 Icd	4.2	3.12	Isocitrate dehydrogenase (NADP)	45.6
PA4464 PtsN	4	1.30	Nitrogen regulatory protein	16.7
PA2015 LiuA	4	2.25	Putative isovaleryl-CoA dehydrogenase	42.2
PA4483 GatA	3.9	1.39	Glutamyl-tRNA(Gln) amidotransferase subunit A	51.9
PA3183 Zwf	3.9	2.06	Glucose-6-phosphate 1-dehydrogenase	55.6
PA3635 Eno	3.8	2.99	Enolase	45.2
PA4560 IleS	3.7	2.99	Isoleucine-tRNA ligase	105.5
PA2624 Idh	3.7	2.57	Isocitrate dehydrogenase (NADP)	81.6
PA3529	3.6	1.98	Alkylhydroperoxide reductase C	21.8
PA0552 Pgk	3.5	1.40	Phosphoglycerate kinase	40.4
PA0130 BauC	3.4	1.39	Putative 3-oxopropanoate dehydrogenase	53.3
PA0382 TrmB	3.4	1.39	tRNA [guanine-*N*(7)-]-methyltransferase	27.6
PA1838 CysI	3.3	2.52	Sulfite reductase	62.1
PA2513 AntB	3.3	1.30	Anthranilate dioxygenase small subunit	19.3
PA3286	3.2	1.33	Beta-ketodecanoyl-(acyl-carrier-protein) synthase	38.2
PA3440	3.2	1.40	Hypothetical	11.6
PA4756 CarB	3	1.56	Carbamoyl-phosphate synthase large chain	117.3
PA1844 Tse1	3	0.85	Known Hcp1 effector	16.4

a Hits are ordered by fold change, which is the difference between the Hcp-FLAG pulldown and the Hcp untagged pulldown. All hits were not enriched in the respective VgrG-FLAG control.

b Significance is −log *P* value, where >1.3 equals a *P* value of <0.05.

c CoA, coenzyme A.

**TABLE 2:** Hcp2-FLAG top interaction hits^*a*^

Protein	Fold change	Significance^*b*^	Description of protein	Size (kDa)
PA2066	10	2.71	Hypothetical, YdcF family protein	23.9
PA4226 PchE	4.4	1.52	Dihydroaeruginoic acid synthetase	156.4
PA2966 AcpP	3.1	1.35	Acyl carrier protein	8.7

a Hits are ordered by fold change, which is the difference between the Hcp-FLAG pulldown and the Hcp untagged pulldown. All hits were not enriched in the respective VgrG-FLAG control.

b Significance is −log *P* value, where >1.3 equals a *P* value of <0.05.

**TABLE 3:** Hcp3-FLAG top interaction hits^*a*^

Protein	Fold change	Significance^*b*^	Description of protein	Size (kDa)
PA0256	8.4	1.76	Hypothetical	33.5
PA3187 GltK	8	1.5	ATP-binding component of ABC transporter	42.2
PA2372	5.8	1.60	Hypothetical	21
PA3076	4.3	1.52	Hypothetical	39.4
PA0381 ThiG	3.9	1.77	Thiazole synthase	28.2
PA1373 FabF2	3.4	1.48	3-Oxoacyl-(acyl-carrier-protein) synthase 2	43.5
PA3182 PgI	3.3	1.87	6-Phosphogluconolactonase	25.6
PA4920 NadE	3	1.65	NH_3_-dependent NAD^+^ synthetase	29.7
PA0771 Era	3	2.10	GTPase	34.5

a Hits are ordered by fold change, which is the difference between the Hcp-FLAG pulldown and the Hcp untagged pulldown. All hits were not enriched in the respective VgrG-FLAG control.

b Significance is −log *P* value, where >1.3 equals a *P* value of <0.05.

10.1128/mBio.00262-21.10 DATA SET S1 Mass spectrometry data for Hcp and VgrG pulldown. Download Data Set S1, XLSX file, 1 MB. Copyright © 2021 Howard et al. 2021 Howard et al. https://creativecommons.org/licenses/by/4.0/ This content is distributed under the terms of the Creative Commons Attribution 4.0 International license.

### PA2066 and PA0256 are putative antibacterial T6SS toxins.

**FIG 3:**
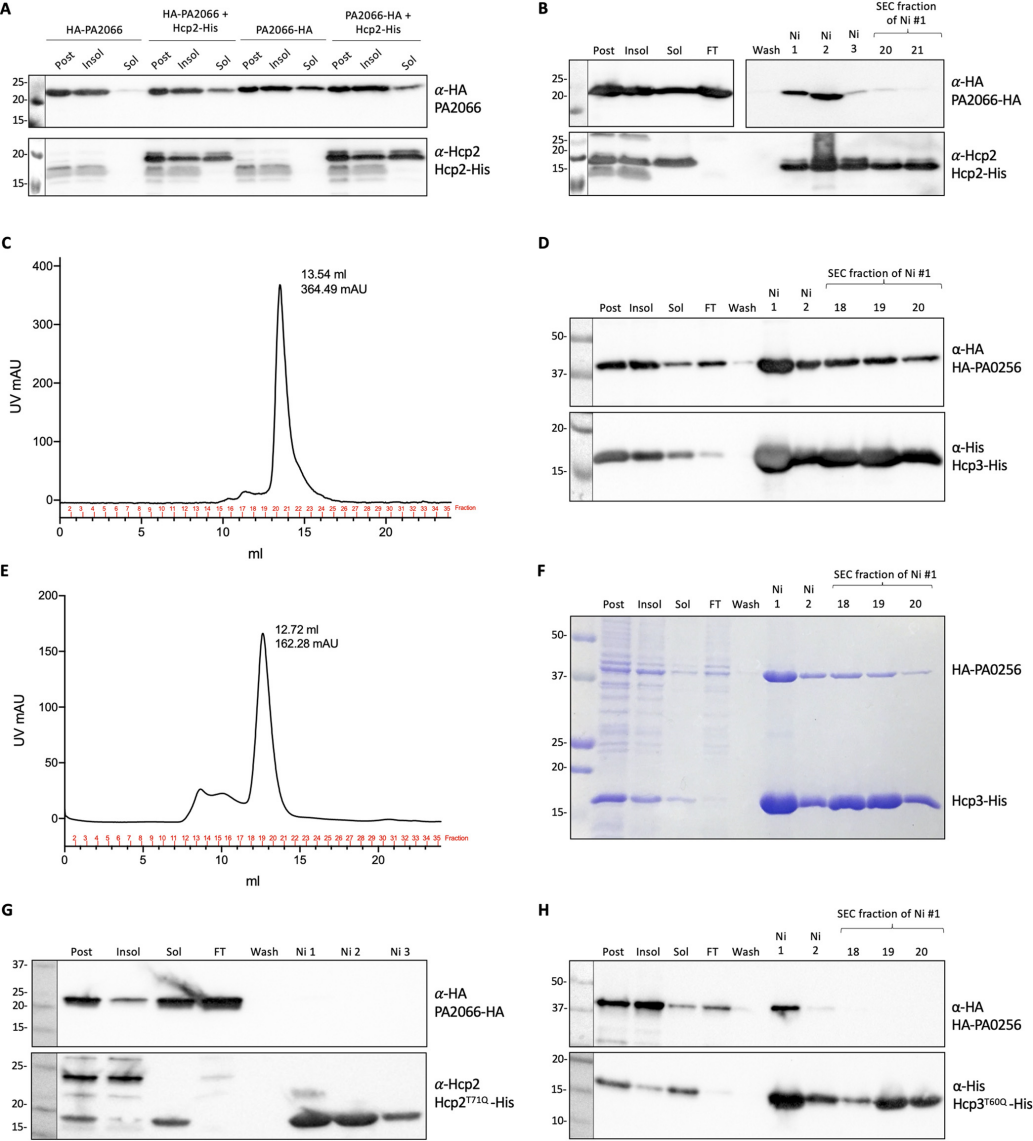
PA2066 and PA0256 are putative Hcp cargoes. (A) Solubility test for PA2066; E. coli BL21 cells expressed either HA-tagged PA2066 alone or with Hcp2-His. After induction, cells were grown at 18°C overnight, harvested, and resuspended in purification resuspension buffer, sonicated, and centrifuged. Western blot of postexpression, insoluble, and soluble samples. (B) Western blot of Hcp2-His and PA2066-HA copurification; lanes labeled at the top are postexpression sample, insoluble and soluble samples after sonication and clarification, flowthrough (FT) from the Ni-NTA column, wash fraction before elution, Ni fractions corresponding to the elution peak, and SEC fractions corresponding to the gel filtration peak. The Hcp2-His with PA2066-HA copurification Western blot membrane was cut so that the wash-SEC fractions could be exposed longer to check that the wash fraction was clear and if there were bands detectable in the SEC peak fractions with anti-HA antibody. (C) SEC chromatograph of purified Hcp2-His and PA2066-HA. Hcp3-His and HA-PA0256 copurification Western blot (D), SEC chromatograph (E), and Coomassie blue stain (F). (G) Copurification of Hcp2^T71Q^-His and PA2066-HA. (H) Copurification of Hcp3^T60Q^-His and HA-PA0256. The antibodies used are labeled on the right: top, anti-HA antibody (BioLegend) at 1:1,000 concentration; bottom, either anti-Hcp2 antibody (Eurogentec) at 1:500 concentration or anti-His antibody (GenScript) at 1:1,000 concentration. Molecular weight standards are on the left.

In contrast to Hcp1, no Hcp-bound effectors for Hcp2 and Hcp3 have been identified to date. For this reason, we investigated the two top hits from the Hcp2 and Hcp3 *in vivo* pulldown, i.e., PA2066 for Hcp2 (10-fold enrichment) and PA0256 for Hcp3 (8.4-fold enrichment). We validated the interaction by performing copurification experiments with C-terminally His-tagged Hcp (see Fig. S5A to D) and N- or C-terminally HA-tagged PA2066/PA0256. PA2066 was more soluble when tagged with HA at the C terminus (Fig. 3A), but both versions coeluted with Hcp2-His from the nickel column (Fig. 3B and C; data not shown for N terminus). The interaction is weak, since the complex was lost after size exclusion chromatography, but PA2066-HA was not retained on the nickel column if not coexpressed with Hcp2-His (see Fig. S6A), which suggests a specific interaction. The N-terminally HA-tagged version of PA0256 formed a robust complex with Hcp3-His (Fig. 3D to F), which was still present after size exclusion chromatography. We conclude that PA2066 and PA0256 interact with their cognate Hcp partners and that the strength of the interaction varies depending on the Hcp-effector pair analyzed. This is also seen for Hcp1 interactions; Tse2 interacts strongly with Hcp1, while other Tse effectors display a weaker affinity.

10.1128/mBio.00262-21.5 FIG S5 Purification of Hcp2 and Hcp3. (A) Coomassie blue stain of Hcp2-His purified via Ni resin and then run through a SD200 SEC column for further clarification. Lanes labeled on top are pre- and postexpression samples, insoluble and soluble samples after sonication and clarification, flowthrough (FT) from the Ni-NTA column, wash fraction before elution, Ni fractions corresponding to the elution peak, and SEC fractions corresponding to the gel filtration peak. Molecular weight standards are on the left. (B) SEC chromatograph of purified Hcp2-His. (C) Coomassie blue stain of Hcp3-His purification. (D) SEC chromatograph of purified Hcp3-His. Download FIG S5, TIF file, 1.1 MB. Copyright © 2021 Howard et al. 2021 Howard et al. https://creativecommons.org/licenses/by/4.0/ This content is distributed under the terms of the Creative Commons Attribution 4.0 International license.

10.1128/mBio.00262-21.6 FIG S6 PA2066 and PA0256 purification controls. PA2066-HA (A) and HA-PA0256 (B) were expressed without Hcp2-His and Hcp3-His, respectively, and samples were run through the Ni-NTA column alone to confirm the proteins do not interact with the Ni resin. Lanes labeled on top are pre- and postexpression samples, insoluble and soluble samples after sonication and clarification, flowthrough (FT) from the Ni-NTA column, wash fraction before elution, and Ni fractions corresponding to the elution peak. The antibodies used are labeled on the right: anti-HA antibody (BioLegend) at 1:1,000 concentration. Molecular weight standards are on the left. Download FIG S6, TIFF file, 0.5 MB. Copyright © 2021 Howard et al. 2021 Howard et al. https://creativecommons.org/licenses/by/4.0/ This content is distributed under the terms of the Creative Commons Attribution 4.0 International license.

The inner ring Hcp1 residues that were important for the interaction with Tse4 (Fig. 1C to E) were examined for their conservation in Hcp2 and Hcp3. T59 in Hcp1 is conserved in Hcp2 and Hcp3 (residues T71 and T60, respectively) and, in all cases, located on the inner ring (see Fig. S7). Hcp2 and Hcp3 substitutions of these residues impacted interaction with the putative effectors PA2066 and PA0256, respectively (compare Fig. 3D and G and Fig. 3D and H), as did the Hcp1 substitution for Tse4, suggesting a conserved role of this threonine residue in effector interaction.

10.1128/mBio.00262-21.7 FIG S7 Conserved residues between Hcp1, Hcp2, and Hcp3 of P. aeruginosa. (A) Protein sequence alignment of Hcp1, Hcp2, and Hcp3, performed with Clustal Omega multiple sequence alignment. The residue conservation color was added in Jalview by percentage identity with a 50% conservation threshold. Dark purple is for conserved residues, light purple is for similar residues; residue number provided at the start and end of each line. Red box highlights the conserved threonine residue studied here. Hcp1 (B), Hcp2 (C), and Hcp3 (D) dimers with conserved inner ring residues colored and labeled: S31, red; H46/32, gray; T59/60/71, yellow; S108/112, dark green; V110/114, cyan; S111/115, light green; G114/118, light pink; T122/139, orange; E123/126, magenta. Surface representation of dimers, constructed in PyMOL, views from inside the ring. Hcp1 PDB 1Y12, Hcp2 PDB 3HE1, Hcp3 Phyre2 prediction. Download FIG S7, TIF file, 1 MB. Copyright © 2021 Howard et al. 2021 Howard et al. https://creativecommons.org/licenses/by/4.0/ This content is distributed under the terms of the Creative Commons Attribution 4.0 International license.

**FIG 4:**
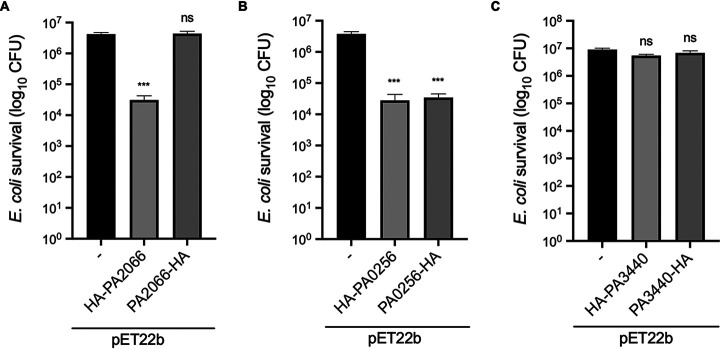
PA2066 and PA0256 are toxic when expressed in E. coli cytoplasm. BL21 cytoplasmic toxicity assay: survival after overnight growth on 100 mM IPTG inducing agar. When grown on 2% glucose inhibiting agar in parallel, all samples grew the same. (A) Toxicity assay of PA2066, *n* = 5; the mean with standard error of the mean (SEM) for pET22b is 4,200,000 ± 503,433, for pET22b:HA-PA2066 is 31,133 ± 11,364, and for pET22b:PA2066-HA is 4,380,000 ± 827,419. (B) Toxicity assay of PA0256, *n* = 4; the mean with SEM for pET22b is 3,775,000 ± 670,044, for pET22b:HA-PA0256 is 27,908 ± 15,459, and for pET22b:PA0256-HA is 34,608 ± 10,347. (C) Toxicity assay of PA3440, *n* = 3; the mean with SEM for pET22b is 9,000,001 ± 1,071,518, for pET22b:HA-PA3440 is 5,455,556 ± 577,457, and for pET22b:PA3440-HA is 6,744,444 ± 1,313,228. Statistical testing was conducted by one-way analysis of variance (ANOVA) with Dunnett multiple-comparison test and family-wise significance and confidence level set to *P* = 0.05; each effector compared to pET22b control. ***, *P* < 0.001; ns, not significant. *n* is number of replicate toxicity assays.

Having shown that both PA2066 and PA0256 are putative Hcp cargoes, we assessed whether they can be putative T6SS antibacterial toxins by overexpressing the proteins in E. coli. A standard toxicity assay was performed by plating overnight-grown bacterial cultures on agar plates containing isopropyl-β-D-thiogalactopyranoside (IPTG) (toxin induction) or glucose (toxin repression), and the CFU was enumerated. These experiments revealed that PA2066 N-terminally HA-tagged is toxic in the E. coli cytoplasm (Fig. 4A). There is a 2-log_10_ drop in survival, equivalent to 135 times less survival when comparing the mean survival of E. coli carrying pET22b to that carrying pET22b/HA-PA2066. When harboring a C-terminal HA tag, PA2066 did not display any toxicity, which suggests that the tag in that position interferes with PA2066 activity. In the case of PA0256, the tag position had no significant impact, since we observed a 2-log_10_ drop in survival, equivalent to 135 and 109 times less survival when comparing the mean survival of E. coli harboring pET22b to that harboring pET22b/HA-PA0256 and pET22b/PA0256-HA, respectively (Fig. 4B). We also tested the toxicity of one of the low-molecular-weight hits of unknown function retrieved from the Hcp1 pulldown, i.e., PA3440, but found no difference in the CFU counts (Fig. 4C). Based on our data, we suggest that PA2066 and PA0256 are bona fide Hcp cargoes and might be H2- and H3-T6SS toxins with antibacterial activity, respectively.

While both proteins are uncharacterized, *in silico* analyses of PA2066 showed it to be a YdcF family protein with <70% identity to orthologues in other species. PA2066 contains a Rossmann-like alpha/beta/alpha sandwich fold which has been linked to cofactor binding. The neighboring gene of *PA2066* is *PA2067*, which encodes a predicted HAD family phosphatase. While not studied experimentally, if PA2067 was the immunity protein of this putative effector, it would suggest a kinase-phosphatase toxin-immunity pair, with PA2066 being a kinase, as Rossmann-like alpha/beta/alpha sandwich folds have been found in a characterized family of kinases (41). Immunity proteins counteracting the enzymatic activity of their cognate toxin have been previously reported, for example, in the case of Tri1 that removes the FtsZ ADP ribose modification driven by the T6SS toxin Tre1 from Serratia proteamaculans (42).

PA0256 contains an uncharacterized DUF4347 domain and is primarily found in P. aeruginosa with the exception of a single strain of Acinetobacter baumannii (99.35% protein identity) and three strains of Pseudomonas fluorescens (60.97, 37.3, and 36.99% protein identity). The genomic organization does not suggest any candidate encoding an immunity protein; however, this effector is encoded in the vicinity of the *vgrG2b* orphan cluster (*PA0259-PA0263*), which still contains genes of unknown function (29).

### Tse4 features that modulate Hcp-dependent delivery.

**FIG 5:**
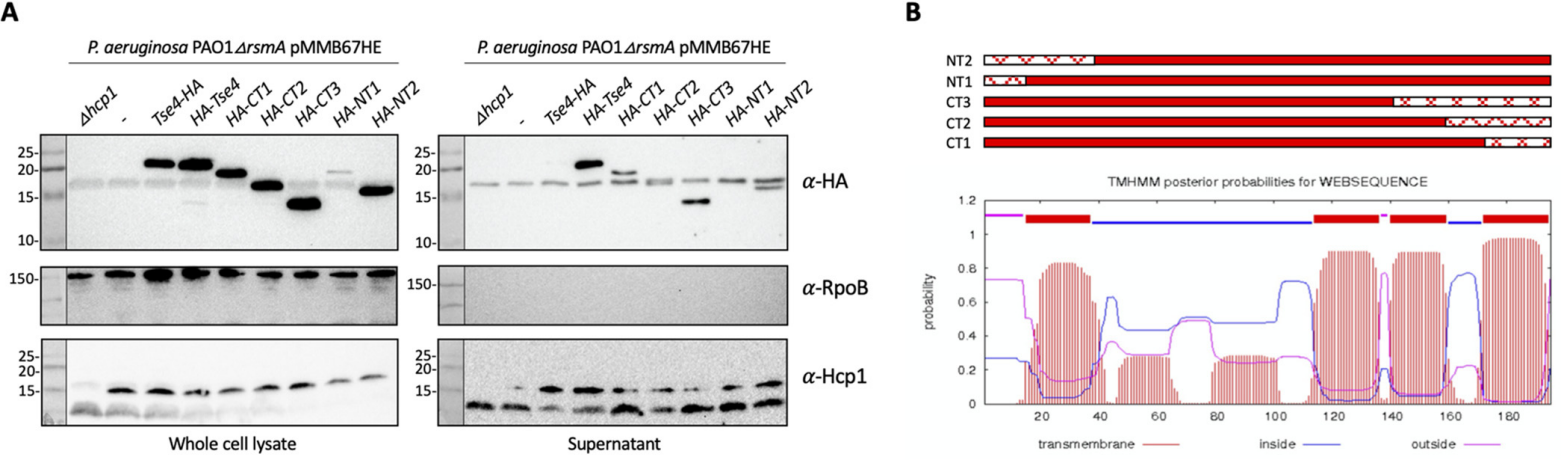
Transmembrane domain truncations of Tse4 are still secreted. (A) Western blot of secretion assay of P. aeruginosa PAO1 Δ*rsmA* strains expressing HA-tagged Tse4 truncations from pMMB67HE. Whole-cell lysate (left) and supernatant (right) harvested 4 h postinduction with 250 μM IPTG. Lanes labeled on the top are the strains. The antibodies used are labeled on the right: top, anti-HA antibody (BioLegend) at 1:1,000 concentration; middle, anti-RpoB antibody (NeoClone) at 1:5,000 concentration used for loading control and cell lysis control; bottom, anti-Hcp1 antibody (Eurogentec) at 1:500 concentration. Molecular weight standards are on the left. Representative blots of three independent experiments. (B) Predicted transmembrane domains of Tse4 (TMHMM server v 2.0), with truncation constructs shown above; solid red is the construct, hatched red is the residues deleted.

We observed that the position at which a T6SS toxin is tagged, for example, addition of an HA tag at the C terminus of Tse4 (Fig. S3C), may interfere with the interaction between the effector and its cognate Hcp and subsequently secretion (Fig. 5A). We used Tse4 to investigate whether specific features of a T6SS-bound toxin might be required for T6SS-dependent delivery of Hcp cargoes and whether N- or C-terminal residues are important for recognition by Hcp. Tse4 is a pore-forming protein (39), and putative transmembrane domains are present near both the N- and C-terminal regions of the protein (Fig. 5B). We engineered truncations of Tse4 still harboring an N-terminal HA tag and assessed secretion of these variants. The intact and various truncated Tse4 constructs were expressed in the H1-T6SS active P. aeruginosa strain PAO1 Δ*rsmA*; while all constructs were expressed, HA-NT1 seemed less stable (Fig. 5A). All other variants were secreted, including those carrying deletions at the C terminus. This indicates that the presence of the tag at the C terminus does not necessarily obscure key residues for Hcp recognition, but instead, the tag may block Hcp interaction by altering the overall fold of the protein. This is supported by our observation that Tse4 toxicity in E. coli was lost for Tse4-HA but not for the HA-Tse4 version (Fig. S3A). We conclude that specific regions or residues of Hcp cargoes, notably within the N- and C-terminal regions, are not a major determinant of Hcp-dependent recognition. Instead, the effector size and the steric fit with key residues within the Hcp lumen are likely two of the main drivers for the loading of effectors into the T6SS tube.

### Large T6SS chimeric effectors block T6SS function when bound to Hcp.

**FIG 6:**
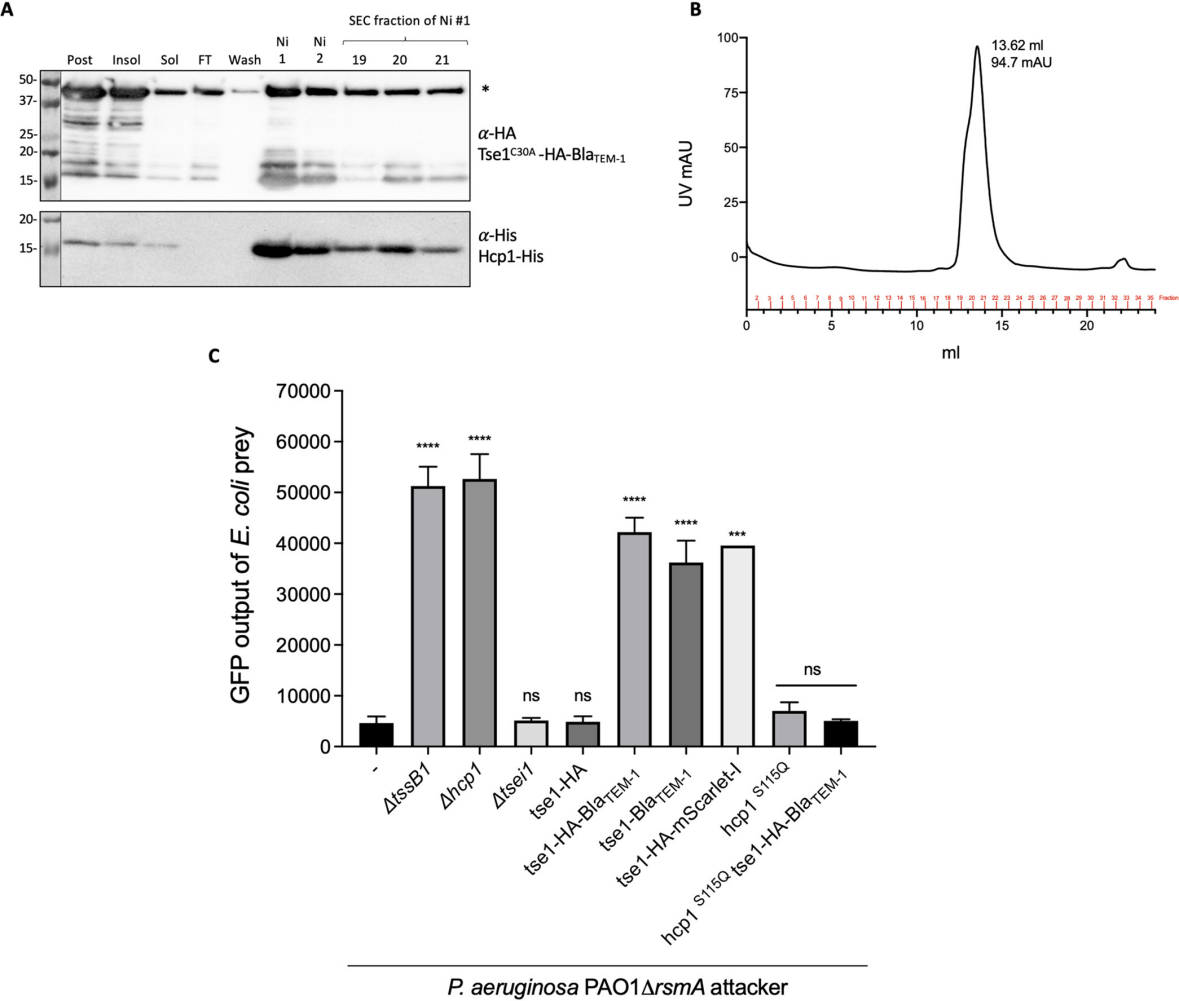
Large Tse1 chimera can bind Hcp1 but block H1-T6SS-dependent killing. (A) Western blot of Hcp1-His and Tse1^C30A^-HA-Bla_TEM-1_ copurification; lanes labeled on the top are postexpression sample, insoluble and soluble samples after sonication and clarification, flowthrough (FT) from the Ni-NTA column, wash fraction before elution, Ni fractions corresponding to the elution peak, and SEC fractions corresponding to the gel filtration peak. The antibodies used are labeled on the right: top, anti-HA antibody (BioLegend) at 1:1,000 concentration; bottom, anti-His antibody (GenScript) at 1:1000 concentration. Molecular weight standards are on the left. Asterisk denotes the correct size of Tse1^C30A^-HA-Bla_TEM-1_ at 46.4 kDa. (B) SEC chromatograph of purified Hcp1-His and Tse1^C30A^-HA-Bla_TEM-1_. (C) Competition assay of P. aeruginosa PAO1 Δ*rsmA* attackers against E. coli-GFP prey after a 13-h competition. GFP output represents the number of E. coli cells in the sample. Statistical testing was conducted by one-way ANOVA with Dunnett multiple-comparison test and family-wise significance and confidence level set to *P* = 0.05; each strain compared to parental P. aeruginosa PAO1 Δ*rsmA* strain. ****, *P* < 0.0001; ***, *P* < 0.001; ns, not significant. Mean and SEMs of *n* biological replicates: *n* = 6, 6, 4, 6, 6, 6, 6, 1, 3, and 3, in order of strains.

We examined whether the addition of a large extension to an Hcp cargo might interfere with its secretion. When using the Hcp1 Tse1 toxin fused with beta-lactamase (Bla), we observed that they copurified and formed a stable complex (Fig. 6A and B). We tested whether the Hcp1-bound chimera was delivered into target cells by performing competition assays using green fluorescent protein (GFP)-tagged E. coli as prey for the P. aeruginosa attacker. The *tssB1* or *hcp1* mutant was less effective at killing E. coli (Fig. 6C), since an 11-fold higher fluorescence level than for the wild type was detected. Deletion of *tse1* did not have a drastic effect on prey killing, since other H1-T6SS-dependent toxins were still delivered. When the *tse1* gene was replaced on the chromosome with the gene encoding the Tse1-Bla chimera, killing was drastically reduced (9-fold fluorescence increase), nearly as much as with the inactive T6SS mutants. The lack of killing is not linked to the presence of a tag at the C terminus, since the addition of a small HA tag (Tse1-HA construct) did not interfere with killing. Instead, the addition of another large protein, mScarlet-I, also abrogated killing. To confirm that a large Tse1 chimera blocks T6SS firing because it binds to Hcp1, we used a chromosomally encoded inner ring mutant of Hcp1, Hcp1^S115Q^, which we previously showed lacks interaction with Tse1 (Fig. S4A). An attacker strain P. aeruginosa PAO1 Δ*rsmA hcp1^S115Q^* was still able to kill E. coli (Fig. 6C), showing that the *hcp1* mutation itself does not stop T6SS activity. Finally, when this strain expressed Tse1-HA-Bla, E. coli was killed to the same level as with the parental attacker, showing that in the absence of interaction between Tse1-HA-Bla and Hcp1^S115Q^, there is no longer blocking of the T6SS.

**FIG 7:**
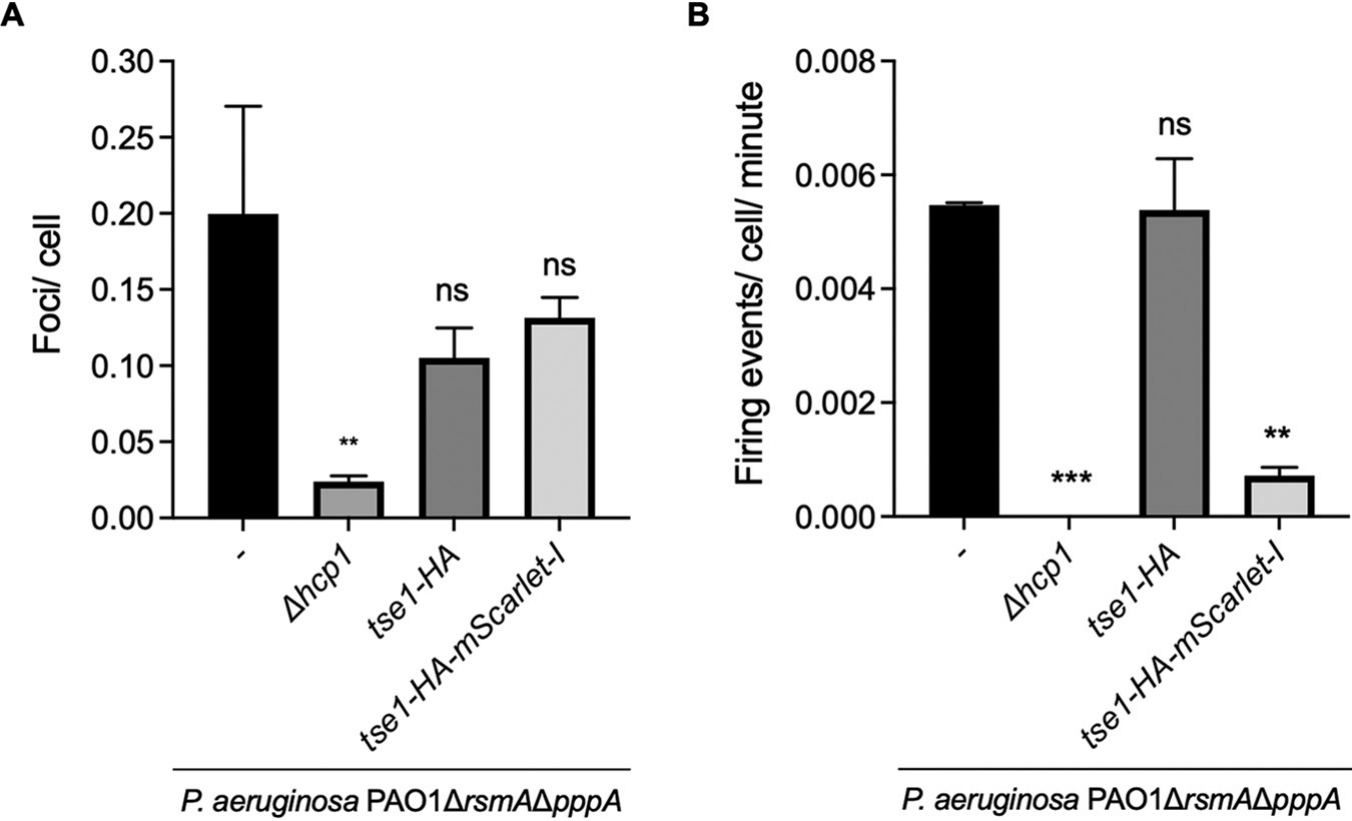
Large Tse1 chimera block H1-T6SS firing. Microscopic analysis of H1-T6SS firing in P. aeruginosa PAO1 Δ*rsmA* Δ*pppA* strains determined through detection of TssB1-sfGFP. (A) Proportion of cells that contain a TssB1-sfGFP focus at one time. (B) Proportion of cells that had an extension and contraction firing event per minute. Means are from two or three independent experiments of ∼40,000 cells with error bars representing the SEMs. P. aeruginosa PAO1 Δ*rsmA* Δ*pppA tssB1-sfGFP* strain total cells, 37,215 from two independent experiments; PAO1 Δ*rsmA* Δ*pppA* Δ*hcp1 tssB1-sfGFP* strain total cells, 31,336 from three independent experiments; PAO1 Δ*rsmA* Δ*pppA tssB1-sfGFP tse1-HA* strain total cells, 40,492 from three independent experiments; PAO1 Δ*rsmA* Δ*pppA tssB1-sfGFP tse1-HA-mScarlet-I* strain total cells, 39,589 from three independent experiments. Statistical testing was conducted by one-way ANOVA with Dunnett multiple-comparison test and family-wise significance and confidence level set to *P* = 0.05; each effector compared to parental P. aeruginosa PAO1 Δ*rsmA* Δ*pppA* strain. ***, *P* < 0.001; **, *P* < 0.01; ns, not significant.

From the above-described experiments, we conclude that unusual Hcp-effector complexes, notably, if the effector is large, may block Hcp ring stacking and therefore extension of the T6SS sheath. This was further validated by monitoring T6SS sheath formation using a P. aeruginosa strain carrying a double *rsmA pppA* mutation and expressing a GFP-tagged version of TssB. The *pppA* mutation results in a larger number of T6SS firing events and allows robust quantification of the number of T6SS sheaths formed in each strain (43). We used fluorescence microscopy to assess the number of T6SS firing events per cell for 1 min for each strain (Fig. 7). Firing events were determined by elongation of fluorescent foci into a sheath and then contraction back into a focus and sometimes disassembly (disappearance of foci). In the parental P. aeruginosa PAO1 Δ*rsmA* Δ*pppA* strain, ∼5.5% of the cells showed a firing event within 1 min, while in the strain lacking *hcp1*, no firing events were observed after examining 31,336 cells. When the Tse1-HA variant was expressed instead of Tse1, there was still ∼5.5% of cells that showed a firing event within 1 min; however, when Tse1-HA-mScarlet-I was expressed, this number decreased to ∼0.7% (∼8-fold fewer firing events). Together, these data show that loading large chimeric effectors onto Hcp1 results in a lack of delivery of the chimera and impairs delivery of any other T6SS effectors by preventing normal assembly and contraction of the T6SS sheath.

### Phylogenetic analysis of Hcp domains does not discriminate specialized versus nonspecialized proteins.

The amino acid sequence of Hcp proteins and their predicted structures are highly conserved. Therefore, it may be hard to identify key residues in the Hcp protein which may account for specific interactions with a given effector. This seems to be true for the S31 residue in Hcp1, which is involved in the interaction with Tse2 but does not play a role in the interaction with Tse1, Tse3, or Tse4 (34, 35). Other inner ring residues, such as S115 and T59, have a broader impact, and substitution of those amino acids results in loss of interaction with all known Hcp cargoes.

**FIG 8:**
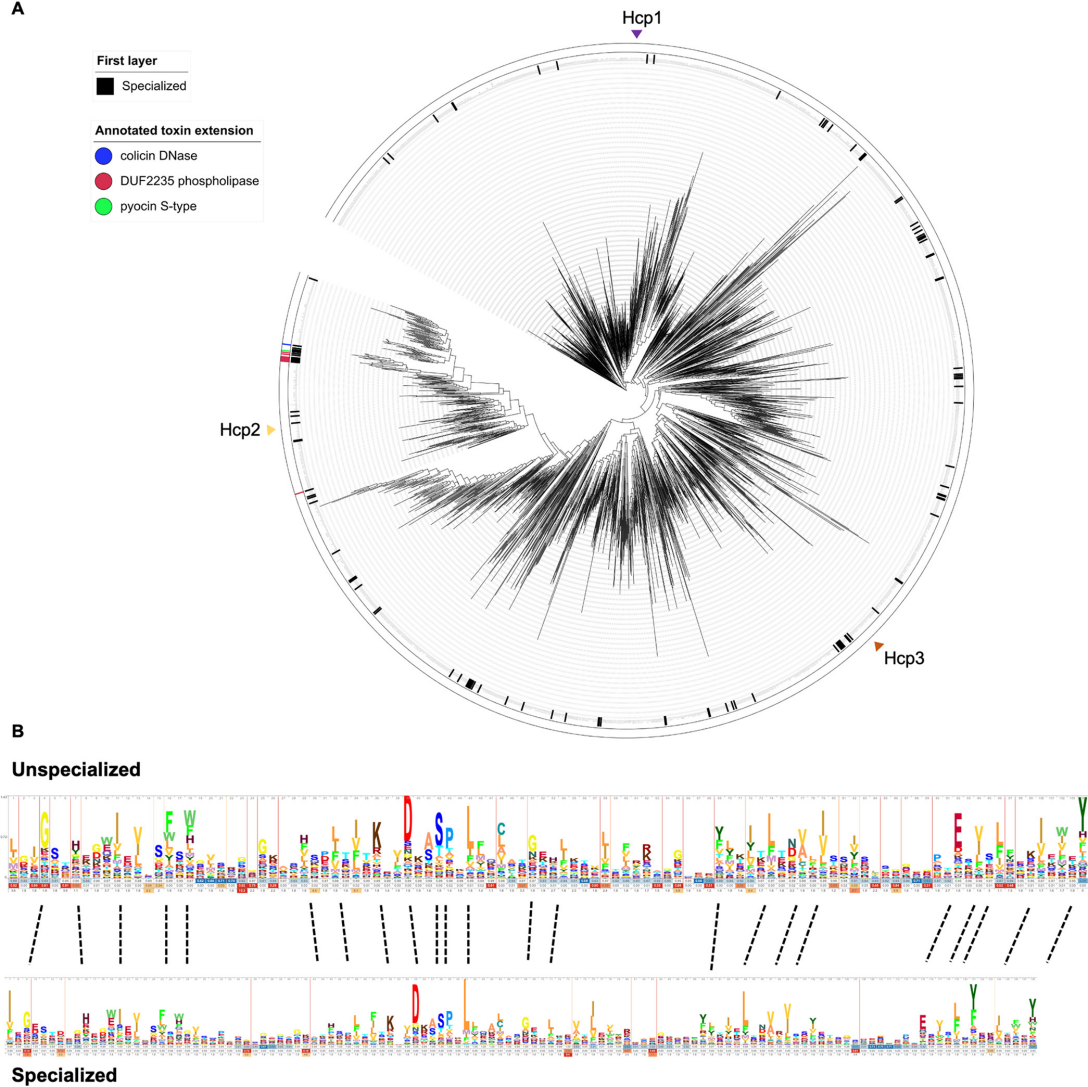
Phylogenetic tree of Hcp and HMM of specialized (evolved) and unspecialized Hcp. (A) The 61,461 Hcp domains were clustered and used to construct the phylogenetic tree. Each leaf represents an Hcp cluster representative. In the first layer, specialized Hcps are colored black. Second layer shows known C-terminal toxic Pfam domains in specialized Hcps. If there is no recognizable effector domain, they are shown without color, and they are colored green, red, or blue if they have a pyocin S-type, DUF2235 phospholipase, or colicin DNase domain, respectively. (B) HMM logo of cluster representatives of specialized and unspecialized Hcps.

In specialized Hcps which contain a C-terminal domain accounting for the toxic activity, specific interaction might not be required. Therefore, if key residues required for cargo recognition existed, they might be unimportant in this context. We constructed a phylogenetic tree using ∼61,461 Hcp proteins retrieved from the Integrated Microbial Genomes (IMG) database (44) (Fig. 8A) and including core Hcps, such as Hcp1 to -3 from P. aeruginosa, but also the core domain of specialized Hcps, such as Hcp-ET-1-5 from *Enterobacteriaceae* (33). The core Hcp domain is ∼160 amino acids long, and so we categorized Hcp proteins with an extra C-terminal extension of >60 amino acids long as specialized. These specialized Hcps are colored in black in the first layer of the tree. The second layer denotes the experimentally validated toxic domains for specialized Hcps (33). As seen from the distribution, there is no clustering of specialized Hcp proteins and core Hcps, which suggests that there are no major differences in sequence or structure between unspecialized and specialized Hcp core domains. Conserved residues in both categories of Hcp core domains do not indicate any obvious differences (Fig. 8B), which mean that those residues are most likely involved in the folding of the proteins and formation of Hcp rings. Overall, this suggests that interaction between cargo effectors and core Hcp domains relies on an overall structural fold and fit within the inner ring rather than on specific motif recognition.

## DISCUSSION

The idea of Hcp being a dual component with structural and effector function is akin to what has been shown for VgrG, which is a puncturing device but also interacts directly with effectors or carries effector extensions. However, there was very little functional demonstration of evolved Hcps, except for a few in E. coli (33). In addition, there were very few effectors identified that directly interact with Hcp, except for Tse1 to -3 from P. aeruginosa (34). Here, by using an *in vivo* pulldown approach, we showed that Hcp-bound proteins are probably as abundant as VgrG-bound effectors. Using 3 distinct Hcps from P. aeruginosa, we pulled down several effector candidates, two of which, PA2066 and PA0256, were validated for their direct interaction with Hcp and displayed antibacterial activity. The prominent role of Hcp as a shuttle for effectors also has important implications for the number of effectors which could be injected by one T6SS device upon a single contraction. Indeed, the trimeric VgrG spike likely carries at most 3 effectors, one per monomer, while one additional effector could be attached to the monomeric PAAR protein; this would result in the delivery of a total of 4 effectors per T6SS expulsion. Considering the sheath wraps around the Hcp stack and expands across the cytoplasm, one would expect more than 100 Hcp rings to be released from each firing event (45). If every ring was loaded with an effector, it becomes obvious that the main T6SS toxic payload is linked with Hcp and not VgrG or PAAR.

One observation we made when performing the pulldown experiments with P. aeruginosa Hcp1, Hcp2, and Hcp3 was that there were no common hits between the lists of identified putative effectors, which suggests a specificity in the Hcp-effector interaction. Also of note is the fact that there seem to be few Hcp2 and Hcp3 effectors compared to the large number of hits pulled down with Hcp1. Sequence and structural comparisons between the 3 Hcp proteins showed they all have the same highly conserved residues, which likely account for their conserved structure. Yet some residues might be described as a key feature for Hcp-effector interaction. For example, a conserved threonine residue within the inner Hcp ring is shown, when substituted for a glutamine, to abrogate interaction between all 3 Hcps and their cognate effectors (34). This of course may not account for specificity but rather for a good steric match between the Hcp and its substrate. In terms of specificity, one key Hcp residue, S31, has been proven to impact Hcp1 interaction with Tse2 only but not with other Hcp1 partners (34). It is obvious that one would need to have a better grasp of how Tse2, and possibly Tse1 and Tse3, fits within the Hcp1 ring to assess how S31 would only impact the interaction with Tse2. The existence of specific residues within these effectors is thus yet to be clearly proven.

Understanding the factors that drive the recognition of a cargo by Hcp is one pending question, while another key question regarding Hcp-dependent delivery is how the effector interacts with Hcp without disturbing its packing, which is crucial for sheath polymerization (46, 47). From previous studies, it is proposed that the effector may just fit into the ring, as for Hcp1-Tse2 (34). The presence of the effector within a Hcp tube has been shown using cryoelectron tomography not only in isolated Hcp rings but within an intact T6SS-like system, called a metamorphosis-associated contractile structure (MACS), in which the presence of the effector Mif1 was detected as a filled density in the structure as opposed to the condition where Mif1 was lacking (15, 48). To match the inner diameter of the ring and not protrude far out, the size of the effector might be a limiting factor. Here, we showed that addition of a small tag on Tse1 does not interfere with secretion, but addition of a large protein of 30 kDa, despite not hindering the interaction of the chimera with Hcp, arrests sheath assembly and secretion, suggesting that Hcp1-Tse1 complexes no longer stack on top of each other. One possibility which cannot be ruled out is that regions of larger effectors protruding from the Hcp lumen may be lodged at the interface between the Hcp ring and the surrounding sheath (49) but may need these domains to be unfolded.

In our study, we have shown that the breadth of Hcp-effector pairs is likely far beyond our current knowledge. Understanding how this partnership occurs specifically and how these assemblies fit into the T6SS sheath would likely call for detailed structural analyses of the Hcp-effector complexes. This is an area that deserves thorough investigation, since Hcp proteins not only are central to the T6SS mechanism and effector delivery but are antigenic (9) and good targets for potential anti-P. aeruginosa vaccine development (50).

## MATERIALS AND METHODS

### Bacterial strains, plasmids, and growth conditions.

The bacterial strains used in this study are listed in Table S1 in the supplemental material. Plasmids used in this study are listed in Table S2. Bacteria were cultured in lysogeny broth (LB) (Miller) or tryptic soy broth (TSB) (Sigma) or on LB agar (Miller) plates. Unless mentioned otherwise, bacteria were grown at 37°C with agitation. Media were supplemented with ampicillin (50 μg/ml) and chloramphenicol (34 μg/ml) for E. coli and carbenicillin (100 μg/ml), streptomycin (2,000 μg/ml), and tetracycline (100 μg/ml) for P. aeruginosa. Isopropyl-β-d-thiogalactopyranoside (IPTG) was used for E. coli at 50, 100, or 500 μM, where stated, and for P. aeruginosa at 250 μM.

10.1128/mBio.00262-21.8 TABLE S1 Escherichia coli and Pseudomonas aeruginosa strains used in this study. Download Table S1, DOCX file, 0.1 MB. Copyright © 2021 Howard et al. 2021 Howard et al. https://creativecommons.org/licenses/by/4.0/ This content is distributed under the terms of the Creative Commons Attribution 4.0 International license.

10.1128/mBio.00262-21.9 TABLE S2 Plasmids used in this study. Download Table S2, DOCX file, 0.1 MB. Copyright © 2021 Howard et al. 2021 Howard et al. https://creativecommons.org/licenses/by/4.0/ This content is distributed under the terms of the Creative Commons Attribution 4.0 International license.

The E. coli DH5α strain was used as the cloning host, and E. coli BL21(λDE3) was for protein expression. P. aeruginosa chromosomal mutants were constructed as previously described (51). pKNG101 was transferred via three-partner conjugation with E. coli CC118 λ*pir* donor and E. coli 1047/pRK2013 helper strains with counterselection by growth on 20% (wt/vol) sucrose.

### Western blot analysis.

SDS-PAGE and Western blot were conducted as previously described (29). Antibodies used in this study are provided in Table 4.

**TABLE 4:** Antibodies used in this study

Antibody^*a*^	Host	Serum	Dilution	Source
Anti-HA	Mouse	Monoclonal	1:1,000	BioLegend
Anti-His_6_	Mouse	Monoclonal	1:1,000	GenScript
Anti-Hcp1	Rabbit	Polyclonal	1:500	Laboratory collection
Anti-Hcp2	Rabbit	Polyclonal	1:500	Laboratory collection
Anti-RpoB	Mouse	Monoclonal	1:5,000	NeoClone
HRP-conjugated anti-mouse	Rabbit	Polyclonal	1:5,000	Sigma-Aldrich
HRP-conjugated anti-rabbit	Goat	Polyclonal	1:5,000	Sigma-Aldrich

a HRP, horseradish peroxidase.

### Protein purification.

To purify or copurify Hcp with potential effectors, Hcp proteins were expressed from pACYCDuet-1 and effectors from pET22b. Cells were grown in 1 liter TSB supplemented with appropriate antibiotics to an optical density at 600 nm (OD_600_) of 0.6, expression from the plasmids was induced with 500 μM IPTG, and cells were grown at 18°C. Cells were harvested and resuspended in 40 ml buffer (50 mM Tris, 500 mM NaCl, 20 mM imidazole, pH 8) supplemented with 1 tablet of cOmplete protease inhibitor cocktail (Roche). The resuspended cell pellet was sonicated, and insoluble and soluble samples were separated by centrifugation (18,000 × *g*, 4°C, 45 min). The supernatant was filtered (0.45 μm) and applied to Ni-NTA resin columns (HisTrap Fast Flow, 5 ml; GE Healthcare) preequilibrated with resuspension buffer, using an ÄKTA prime plus system (GE Healthcare). The column was washed with 150 ml resuspension buffer before elution with 40 ml elution buffer (50 mM Tris, 500 mM NaCl, 400 mM imidazole, pH 8). Size exclusion chromatography (SEC) of the eluted fractions corresponding to the peak was performed using a Superdex 200 10/300 column (GE Healthcare) preequilibrated in 50 mM Tris, 150 or 250 mM NaCl, pH 8 (SEC of Hcp proteins alone and of Hcp1-His with HA-Tse2 was performed using 250 mM NaCl, whereas SEC of other Hcp-effector combinations was performed using 150 mM NaCl). Presence of both Hcp and effector was assessed by Coomassie stain and Western blot analysis.

### Electron microscopy and image processing.

Ten microliters of the Hcp1 sample diluted to 0.01 mg/ml was deposited onto glow-discharged copper 400 mesh grids with a continuous carbon film (Agar Scientific). After incubation for 2 min, the sample was washed twice with water before being stained with 2% (wt/vol) uranyl acetate for 1 min. Two hundred micrographs were collected with a 4 k by 4 k TVIPS camera on an FEI CM200 microscope operating at 200 kV at a magnification of 30,000× with a defocus range of −1.5 to −3.0 μm. All subsequent data processing steps were performed with crypSPARC v3.0.1 (52). The contrast transfer function parameters for each micrograph were calculated with CTFFIND4 (53). Approximately 1,500 particles were manually picked to generate representative two-dimensional (2D) class averages for automated particle picking of the entire data set. A total of 85,598 particles were autopicked and extracted using a 256- by 256-pixel-size box. After several rounds of 2D classification, 56,350 particles were kept in good classes showing top and side views.

### Bacterial toxicity assays.

E. coli BL21(λDE3) cells expressing effectors from pET22b and Hcp proteins from pACYCDuet-1 were grown in LB supplemented with appropriate antibiotics and 2% glucose. Cells were normalized to an OD_600_ of 1 before serial dilution in phosphate-buffered saline (PBS). Twenty microliters of each dilution was spotted in triplicates on LB agar containing the appropriate antibiotics and inducer (50 μM IPTG for Tse4 and 100 μM IPTG for pulldown hits) or repressor (1% glucose for Tse4 and 2% glucose for pulldown hits). Plates were grown overnight, and CFU were quantified. The number of biological replicates is given in each figure legend.

### Bacterial competition assays.

For P. aeruginosa competition assays against E. coli Top10/pRL662.5:*gfp*, overnight cultures in LB were washed with PBS and normalized to an OD_600_ of 3. One hundred microliters of each P. aeruginosa attacker and E. coli prey were mixed at a 1:1 ratio. Five microliters of the attacker-prey mix was spotted on LB agar, dried, and incubated for 13 h. CFU of the input bacteria were checked to confirm the same numbers of prey and attacker were in each competition. After incubation, competition spots were scraped into 1 ml PBS, resuspended, and serially diluted. Triplicate serial dilutions were spotted on LB agar plates and grown overnight. The 10^−2^ dilution spots were scraped into 1 ml PBS and resuspended. The GFP fluorescence intensity in each triplicate competition spot, which represents the number of E. coli/pRL662.5:*gfp* present, was measured using a microplate reader (BMG Labtech). The number of biological replicates is given in each figure legend.

### FLAG-bead pulldown.

P. aeruginosa PAO1 Δ*rsmA* strains expressing Hcp1/2/3-FLAG, Hcp1/2/3 (no tag), and VgrG1b/2b/3-FLAG from pME6032 were grown overnight in TSB supplemented with 100 μg/ml tetracycline and then subcultured to OD_600_ of 0.1 in TSB supplemented with 50 μg/ml tetracycline. After growth to OD_600_ of 0.3, expression was induced with 250 μM IPTG. For the H1-T6SS proteins (Hcp1-FLAG, Hcp1, and VgrG1b-FLAG), cultures were grown at 37°C for 5.5 h. For the H2-T6SS proteins (Hcp2-FLAG, Hcp2, and VgrG2b-FLAG), cultures were grown at 25°C for 13 h. For the H3-T6SS proteins (Hcp3-FLAG, Hcp3, and VgrG3-FLAG), cultures were grown at 25°C for 24 h. Cultures at an OD_600_ of 200 were harvested by centrifugation (4,000 × *g*, 4°C, 20 min), and the pellet was resuspended in 50 ml PBS. The cells were cross-linked using dimethyl 3,3′-dithiobispropionimidate (DTBP) (Thermo Fisher Scientific) to a final concentration of 5 mM, and the mix was incubated at room temperature for 45 min. Tris-HCl (pH 7.6) was added to a final concentration of 20 mM for 15 min to quench the remaining DTBP. The cross-linked cells were centrifuged (4,000 × *g*, 4°C, 20 min), washed with PBS, and centrifuged. The pellet was resuspended in 35 ml PBS and sonicated. This sample was centrifuged (18,000 × *g*, 4°C, 45 min) to separate the soluble and insoluble proteins, and the supernatant was collected. Forty microliters anti-FLAG M2 magnetic beads (Millipore) (prewashed in PBS) was added to each supernatant and mixed with gentle agitation at 4°C for 4 h to overnight. Beads and bound protein were separated from unbound protein using a magnetic rack by carrying out 8 washes in 1 ml PBS. The beads and bound protein were resuspended in 20 μl PBS and frozen using liquid nitrogen. Three independent experiments for each pulldown were performed.

### Mass spectrometry analysis.

Mass spectrometry analysis was conducted by the Plateforme Protéomique Structurale et Fonctionnelle at the Institut Jacques Monod, Paris. Briefly, samples underwent on-bead digestion with 12.5 mg/ml sequencing-grade trypsin (Promega) before peptide analysis on an Orbitrap Fusion Tribrid mass spectrometer coupled to an Easy-spray nanoelectrospray ion source and an Easy nano-LC Proxeon 1000 liquid chromatography system (Thermo Scientific). Chromatographic separation of the peptides was achieved by an Acclaim PepMap 100 C_18_ precolumn and a PepMap-RSLC Proxeon C_18_ column at a flow rate of 300 ml/min. The solvent gradient consisted of 95% solvent A (water, 0.1% [vol/vol] formic acid) to 35% solvent B (100% acetonitrile, 0.1%[vol/vol] formic acid) over 98 min. An Orbitrap mass spectrometer analyzed the peptides in full ion scan mode, with the resolution set at 120,000 with a *m/z* mass range of 350 to 1550. High energy collision-induced dissociation activation with a collisional energy of 28% permitted fragment acquisition with the quadruple isolation width of 1.6 Da. The linear ion trap was employed in top-speed mode to acquire the tandem mass spectrometry (MS/MS) data with a 50-s dynamic exclusion and a 1-min repeat duration. Maximum ion accumulation times were set to 250 ms for MS acquisition and 60 ms for MS/MS acquisition in parallelization mode.

### (i) MaxQuant analysis.

Data were processed using MaxQuant version 1.5.8.3 (54). Peptides were identified from MS/MS spectra searched against the UniProt P. aeruginosa reference proteome (proteome identifier [ID] UP000002438) (accessed May 2018) using the Andromeda search engine (55). Methionine oxidation and N-terminal acetylation were specified as variable modifications. *In silico* digest of the reference proteome was performed using the “Trypsin/P” setting with up to two missed cleavages allowed. The false discovery rate (FDR) was set at 0.01 for peptides, proteins, and sites. Both the “re-quantify” and “match between runs” functions were enabled. The sequence decoy mode used was “revert.” Protein quantification was performed using the MaxLFQ algorithm within MaxQuant (56). Unique and razor peptides were used for quantification. All other parameters were used as preset in MaxQuant.

### (ii) Perseus analysis.

Data were analyzed using Perseus version 1.5.8.5 (54). Proteins present in the “reverse,” “only identified by site,” and “potential contaminant” databases were removed, and proteins identified by one or more unique peptides retained for further analysis. Label-free quantitation (LFQ) intensities were logarithmized (log_2_), and biological replicates were grouped together before the data were filtered to retain only proteins that appeared in 2 or more biological replicates from at least one sample. Missing log_2_ LFQ intensities were imputed using a downshifted normal distribution (1.8 downshift, 0.3 width). For identification of proteins enriched in Hcp-FLAG immunoprecipitations compared to that in untagged controls, two-sample *t* tests were used (FDR = 0.05). Proteins were considered to be Hcp1-, Hcp2-, or Hcp3-specific interacting partners if they showed a mean log_2_ LFQ intensity difference of ≥+1.5 (with a −log_10_ *P* value of ≥1.3 [equal to *P* ≤ 0.05]) compared to that for the untagged control and were not found to be enriched in a control immunoprecipitation performed using a FLAG-tagged version of the Hcp’s cognate VgrG (Hcp1, VgrG1b; Hcp2, VgrG2b; Hcp3, VgrG3) using the same cutoff. Isoelectric point data for the putative Hcp1-3 cargo proteins were retrieved from https://www.pseudomonas.com (database version 20.2 [2020-09-21]) (57). Proteome-wide isoelectric point data for P. aeruginosa, calculated using the EMBOSS method, were retrieved from http://isoelectricpointdb.org/ (58, 59).

### Secretion assays.

P. aeruginosa PAO1 expressing Tse4 variants from pMMB67HE were grown overnight in TSB supplemented with 100 μg/ml carbenicillin and then subcultured to an OD_600_ of 0.1 in 25 ml TSB supplemented with 50 μg/ml carbenicillin. Strains were grown to an OD_600_ of 0.3 for induction with 250 μM IPTG and then grown a further 4 h. Cells at an OD_600_ of 1 were harvested for the whole-cell lysate sample and resuspended in 100 μl Laemmli buffer. The supernatant was clarified by centrifugation (4,000 × *g*, 4°C, 10 min), and proteins were precipitated with 10% trichloroacetic acid (TCA), washed with 90% acetone, air dried, and resuspended to an OD_600_ of 10 in Laemmli buffer. Whole-cell lysate and supernatant samples were assessed by SDS-PAGE and Western blotting.

### Microscopy.

P. aeruginosa strains were grown overnight in TSB and then subcultured to an OD_600_ of 0.1 in TSB and grown for 5 h. One microliter of each culture was spotted onto a glass-bottomed 35-mm petri dish (μ-Dish 35 mm, high glass bottom; Ibidi) and covered with a 1% (wt/vol) UltraPure agarose (Invitrogen) (dissolved in PBS) pad. Imaging was performed using an Axio Observer Z1 (Zeiss) inverted widefield microscope with a Plan-Apochromat 100×, 1.4 oil Ph3 M27 lens objective (Zeiss), a SpectraX light-emitting diode (LED) light engine (Lumencore), an ORCA-Flash 4.0 digital complementary metal oxide semiconductor (CMOS) camera (Hamamatsu), and an environmental control system and microscope chamber set to 37°C. Phase contrast and fluorescence (exposure time of 200 ms for superfolder GFP [sfGFP]) images were taken to calculate the total cells and number of foci, followed by a fluorescence time-series with images acquired every 2 s for a total of 180 s. Microscopy was performed at Facility for Imaging by Light Microscopy (FILM) Imperial College London. Microscopy image analysis was performed in FIJI ImageJ version 2.1.0/1.53c (60). “Threshold” was used to highlight cells and then “Analyze Particles” was used to count the cells in each image. The “Multi-point” tool was used to manually count the number of foci in an image. Bleach correction was applied to time-series experiments; each extension-contraction event was counted manually using the Multi-point tool, and the final number of events per minute was calculated.

### Bioinformatic analysis.

Statistical analyses were undertaken in GraphPad Prism 8 version 8.4.3. Hcp1 to -3 sequence alignment was performed using Clustal Omega multiple sequence alignment, and sequence conservation was assessed and displayed in Jalview version 2.10.1. (61) using percentage identity with the conservation threshold set to 50%. Phyre2 (62) was used to predict the structure of Hcp3. Hcp1 to -3 protein structures were analyzed and annotated in PyMOL molecular graphics system.

### Phylogenetic tree construction.

All genes with Hcp Pfam domain (PF05638) were collected from the IMG database (44) from Gram-negative bacteria and metagenomes (∼267,000 genes were collected). Hcps found in small metagenomic scaffolds of <5 kb were removed as well as genes containing the PEP-CTERM sorting signal (PF07589). Additionally, Hcp domains shorter than 70 amino acids were also removed. Based on the Hcp hidden Markov model (HMM) (PF05638), Hcp envelope domain boundary was determined using HMMER and then clustered at 60% identity and 80% length using CD-HIT (63) into 2,340 unique clusters in order to prevent redundancy. Cluster representatives were aligned using Clustal Omega (64), and the phylogenetic tree was constructed using FastTree (65). The phylogenetic tree was visualized using iTOL (66).

### HMM’s of specialized and unspecialized Hcps.

Cluster representatives used for the construction of the phylogenetic tree were divided into representatives that came from a specialized Hcp (2,226 cluster representatives) and unspecialized Hcp (114 cluster representatives). These were aligned using Clustal Omega, and each HMM was made and visualized using Skylign (67).
