# Supplementary figures and images for: The Breadth and Molecular Basis of Hcp-Driven Type VI Secretion System Effector Delivery
Source: mBio. 2021 Jun 1;12(3):e00262-21. doi: 10.1128/mBio.00262-21 (PMC8262886; doi:10.1128/mBio.00262-21)

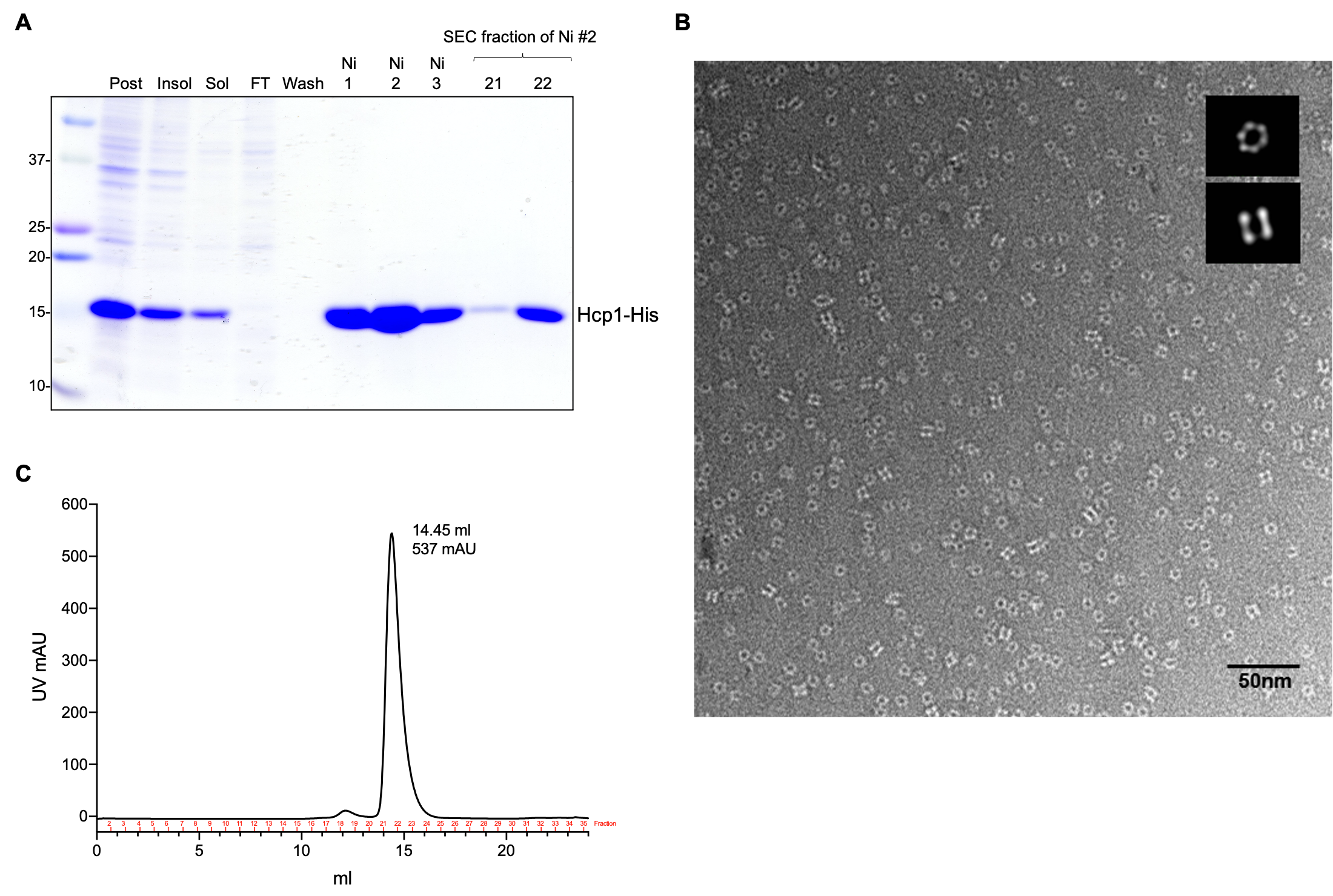

Supplement: FIG S1 [file mbio.00262-21-sf001.tif]

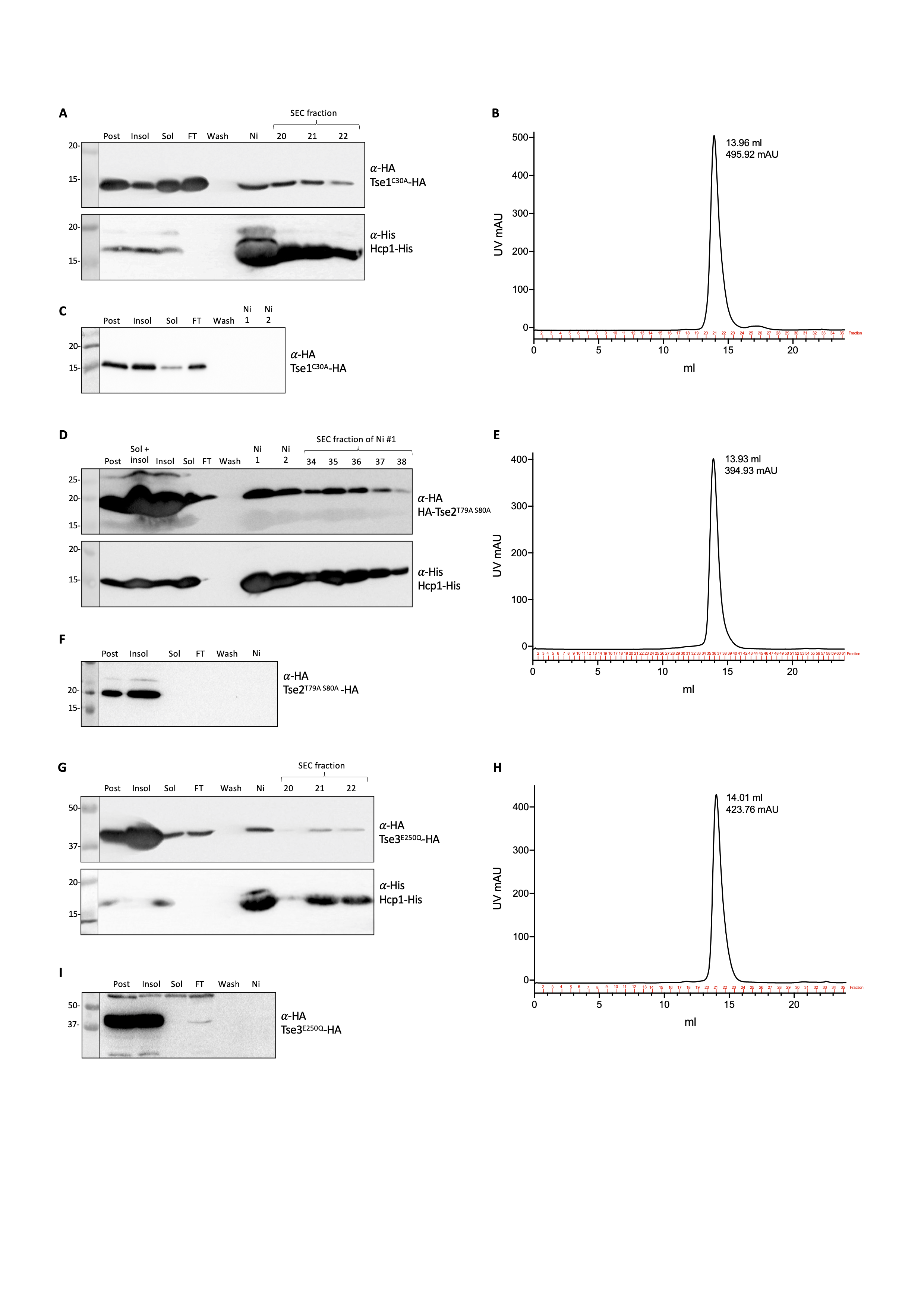

Supplement: FIG S2 [file mbio.00262-21-sf002.tiff]

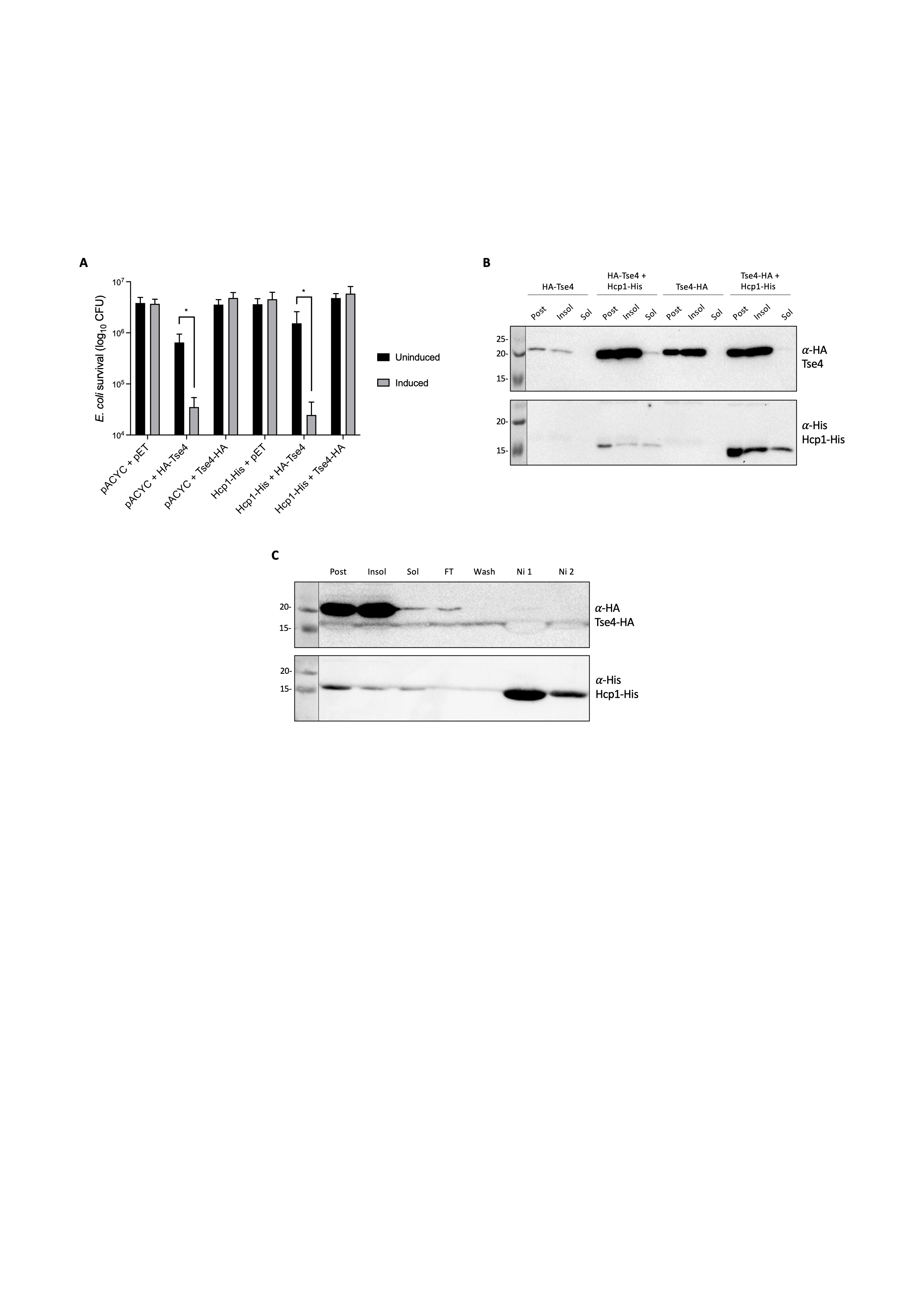

Supplement: FIG S3 [file mbio.00262-21-sf003.tiff]

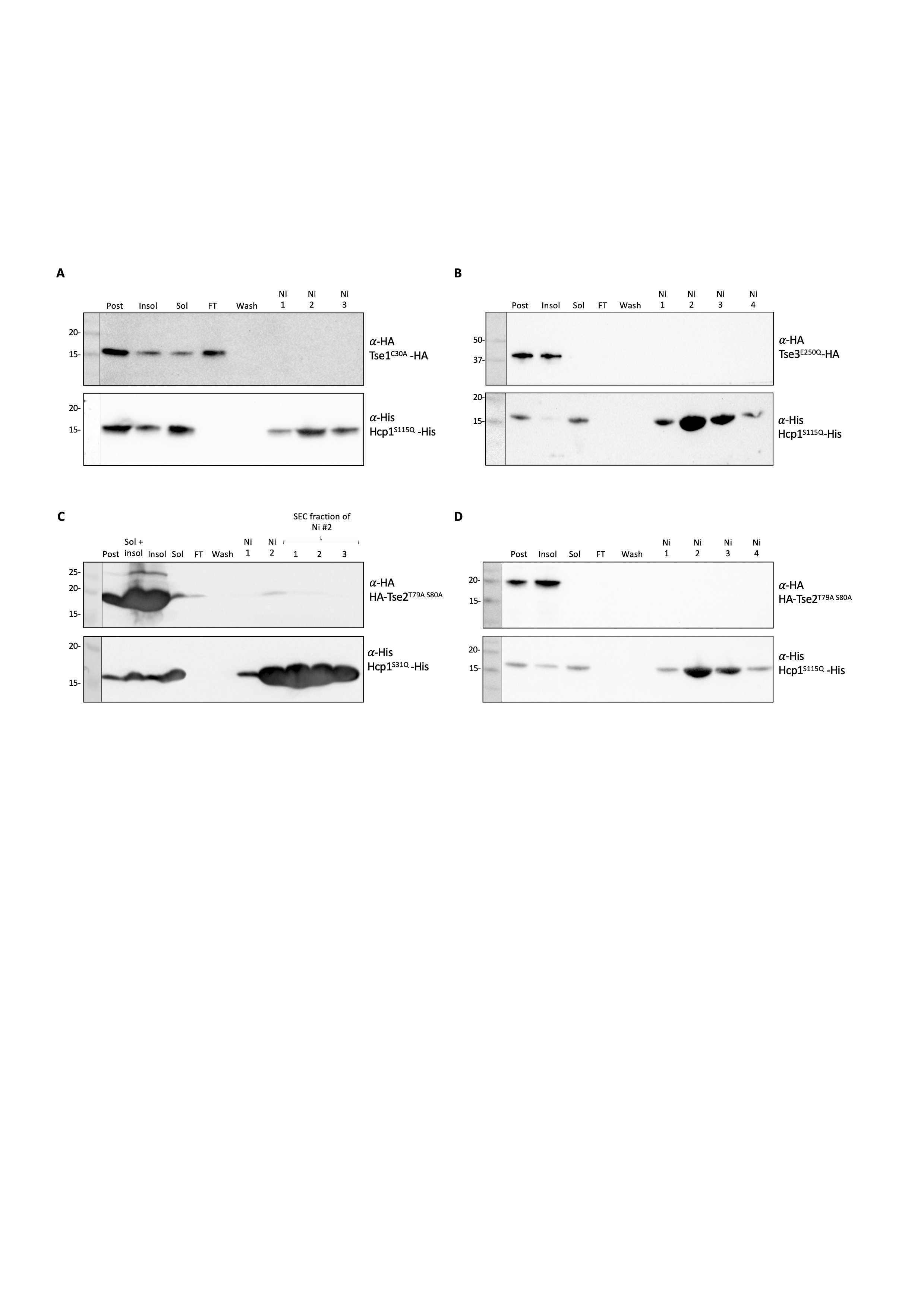

Supplement: FIG S4 [file mbio.00262-21-sf004.tiff]

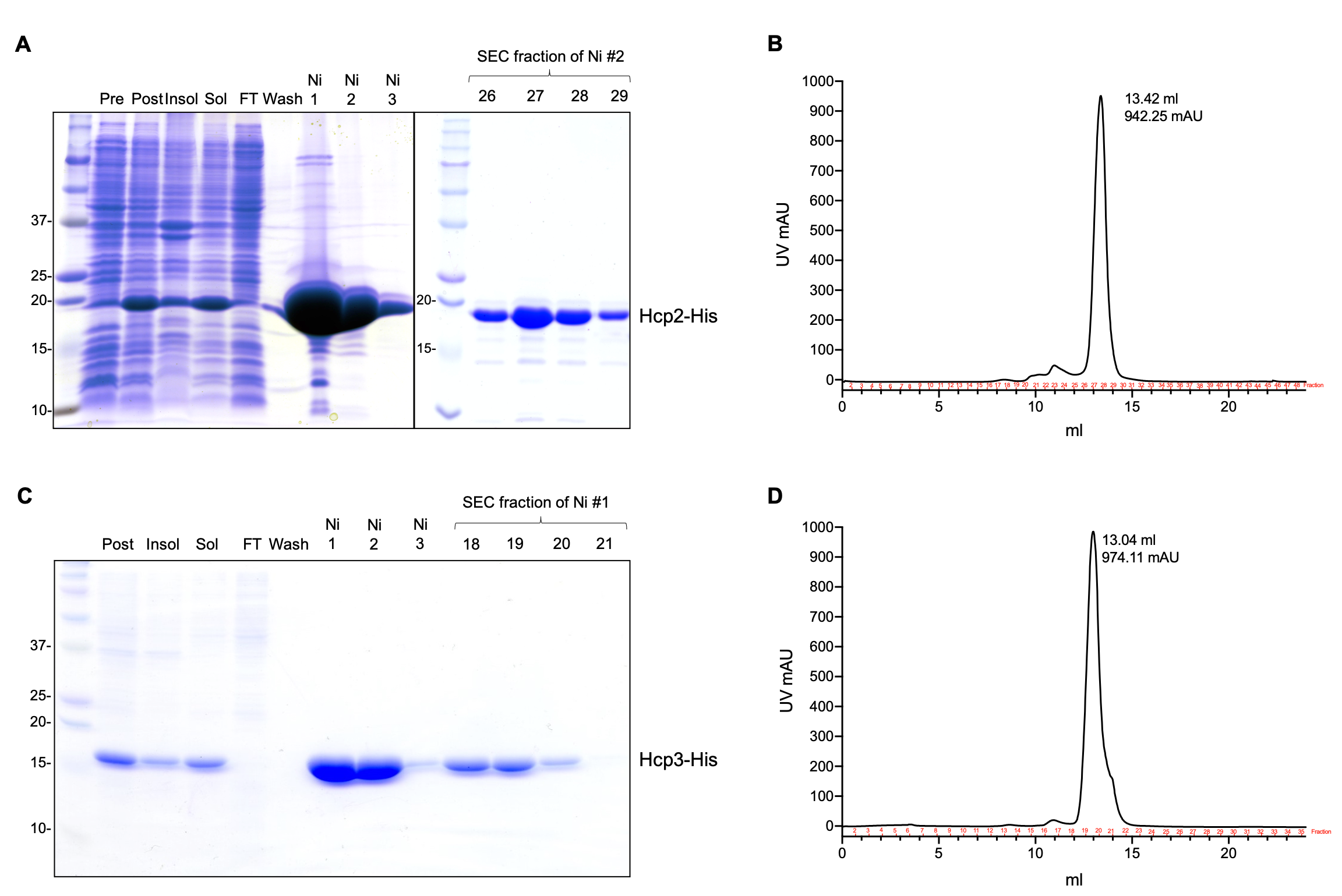

Supplement: FIG S5 [file mbio.00262-21-sf005.tif]

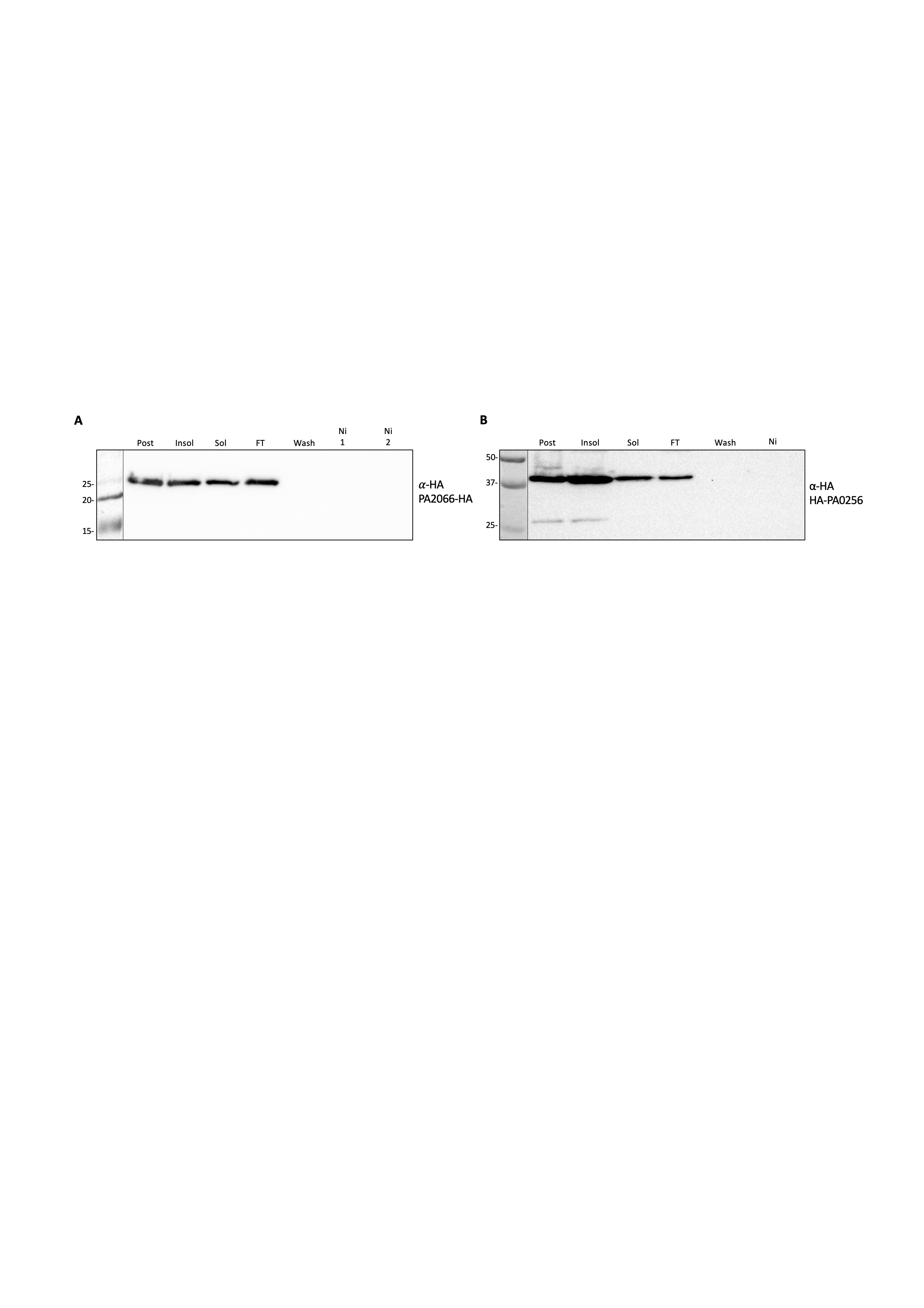

Supplement: FIG S6 [file mbio.00262-21-sf006.tiff]

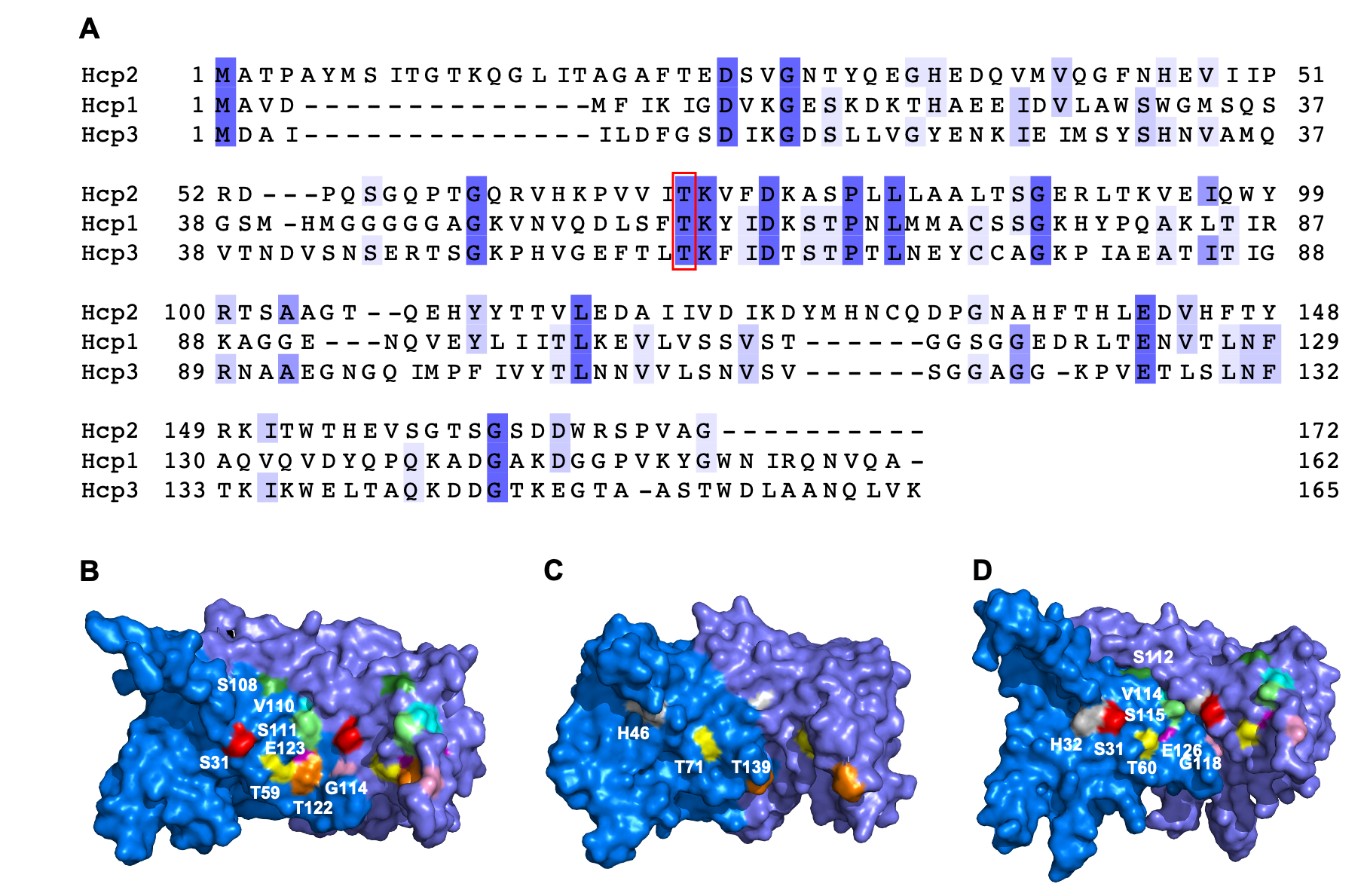

Supplement: FIG S7 [file mbio.00262-21-sf007.tif]
