# Supplementary material for: The Breadth and Molecular Basis of Hcp-Driven Type VI Secretion System Effector Delivery
Source: mBio. 2021 Jun 1;12(3):e00262-21. doi: 10.1128/mBio.00262-21 (PMC8262886; doi:10.1128/mBio.00262-21)
Supplement: TABLE S1 [file mbio.00262-21-st001.docx]

**Table S1 *Escherichia coli* *and Pseudomonas aeruginosa* strains used in this study**

| **Species** | **Relevant features** | **Reference** |
| --- | --- | --- |
| ***E. coli*** |  |  |
| DH5α | Strain used for cloning. F^–^ *endA1 glnV44 thi-.* *1 recA1 relA1 gyrA96 deoR nupG purB20* φ80d*lacZ*ΔM15 Δ(*lacZYA-argF*)U169, hsdR17(*r_K_^-^m_K_^+^*), λ^-^. | Invitrogen |
| CC118*λpir* | Strain used for pKNG101 cloning and replication. Δ(*ara-leu*) *araD ΔlacX74 galE galK-phoA20 thi-1 rpsE rpoB argE* (Ap^R^) *recA1 Rfr λpir.* | (1) |
| 1047 | Helper strain used for three-partner conjugation. (Km^R^), *oriColE1 RK2^-^ Mob^+^ RK2^-^Tra^+^.* | (2) |
| BL21 (λDE3) | Strain used for protein expression. F^–^ *ompT gal dcm lon hsdS_B_*(r_B_–m_B_–) λ(DE3 [*lacI lacUV5-*  *T7p07 ind1 sam7 nin5*]) [*malB*^+^]_K-12_(λ^S^). | Laboratory collection |
| ***P. aeruginosa*** | | |
| PAO1Δ*rsmA* | Deletion of *rsmA* (*PA0905*) to increase T6SS activity, parental used for all experiments, Lausanne subline | Laboratory collection |
| PAO1Δ*rsmA*Δ*tssB1* | Deletion of *rsmA* (*PA0905*) and *tssB1* (*PA0083*) to create a H1-T6SS non-functional mutant | Laboratory collection |
| PAO1Δ*rsmA*Δ*hcp1* | Deletion of *rsmA* (*PA0905*) and *hcp1* (*PA0085*) to create a H1-T6SS non-functional mutant | Laboratory collection |
| PAO1Δ*rsmA*Δ*tsei1* | Deletion of *rsmA* (*PA0905*) and toxin and immunity pair *tse1* (*PA1844*) and *tsi1* (*PA1845*) | This study |
| PAO1Δ*rsmA* *tse1-HA* | Deletion of *rsmA* (*PA0905*) and C-terminal HA-tag on *tse1* (*PA1844*) on the chromosome | This study |
| PAO1Δ*rsmA* *tse1-bla_TEM-1_* | Deletion of *rsmA* (*PA0905*) and C-terminal *bla_TEM-1_* on *tse1* (*PA1844*) on the chromosome | This study |
| PAO1Δ*rsmA* *tse1-HA-bla_TEM-1_* | Deletion of *rsmA* (*PA0905*) and C-terminal HA-tag and *bla_TEM-1_* on *tse1* (*PA1844*) on the chromosome | This study |
| PAO1Δ*rsmA tse1-HA-mScarlet-I* | Deletion of *rsmA* (*PA0905*) and C-terminal HA-tag and *mScarlet-I* on *tse1* (*PA1844*) on the chromosome | This study |
| PAO1Δ*rsmA hcp1^S115Q^* | Deletion of *rsmA* (*PA0905*) and S115Q point mutation on *hcp1* (*PA0085*) on the chromosome | This study |
| PAO1Δ*rsmA hcp1^S115Q^ tse1-HA-bla_TEM-1_* | Deletion of *rsmA* (*PA0905*), S115Q point mutation on *hcp1* (*PA0085*) on the chromosome and HA-tag and *bla_TEM-1_* on *tse1* (*PA1844*) on the chromosome | This study |
| PAO1Δ*rsmA*Δ*pppA tssB1-sfGFP* | Deletion of *rsmA* (*PA0905*) and *pppA* (*PA0075*), C-terminal *superfolder (sf)* *GFP* on *tssB1* (*PA0083*) for microscopy | This study |
| PAO1Δ*rsmA*Δ*pppA*Δ*hcp1 tssB1-sfGFP* | Deletion of *rsmA* (*PA0905*), *pppA* (*PA0075*) and *hcp1* (*PA0085*), C-terminal *superfolder (sf)* *GFP* on *tssB1* (*PA0083*)*,* H1-T6SS non-functional mutant for microscopy | This study |
| PAO1Δ*rsmA*Δ*pppA tse1-HA tssB1-sfGFP* | Deletion of *rsmA* (*PA0905*) and *pppA* (*PA0075*), C-terminal *superfolder (sf)* *GFP* on *tssB1* (*PA0083*)*,* C-terminal HA-tag on *tse1* (*PA1844*) for microscopy | This study |
| PAO1Δ*rsmA*Δ*pppA tse1-HA-mScarlet-I tssB1-sfGFP* | Deletion of *rsmA* (*PA0905*) and *pppA* (*PA0075*), C-terminal *superfolder (sf)* *GFP* on *tssB1* (*PA0083*)*,* C-terminal HA-tag and *mScarlet-I* on *tse1* (*PA1844*) for microscopy | This study |

1. Herrero M, de Lorenzo V, Timmis KN. 1990. Transposon vectors containing non-antibiotic resistance selection markers for cloning and stable chromosomal insertion of foreign genes in gram-negative bacteria. J Bacteriol 172:6557-67.

2. Figurski DH, Helinski DR. 1979. Replication of an origin-containing derivative of plasmid RK2 dependent on a plasmid function provided in trans. Proc Natl Acad Sci U S A 76:1648-52.
