# Supplementary material for: The Breadth and Molecular Basis of Hcp-Driven Type VI Secretion System Effector Delivery
Source: mBio. 2021 Jun 1;12(3):e00262-21. doi: 10.1128/mBio.00262-21 (PMC8262886; doi:10.1128/mBio.00262-21)
Supplement: TABLE S2 [file mbio.00262-21-st002.docx]

**Table S2 Plasmids used in this study**

| **Plasmid** | **Characteristics** | **Source** |
| --- | --- | --- |
| **Cloning vectors** | | |
| pCR™-Blunt II-TOPO™ | Sub-cloning vector for constructs synthesized by KOD PCR, (Km^R^) | Invitrogen |
| pRK2013 | Self-transmissible helper plasmid for three-partner conjugations, Km^R^ | (1) |
| **Chromosomal mutagenesis vectors** | | |
| pKNG101 | Non-replicative suicide vector for *P. aeruginosa* chromosome mutagenesis, *ori6K*, *mobRK2,* *sacB* gene for sucrose sensitivity, Sm^R^ | (2) |
| pKNG101: Δ*hcp1* | Suicide vector to delete *hcp1* (*PA0085*) from *P. aeruginosa*, Sm^R^ | Laboratory collection |
| pKNG101: Δ*pppA* | Suicide vector to delete *pppA* (*PA0075*) from *P. aeruginosa*, Sm^R^ | Laboratory collection |
| pKNG101: Δ*tsei1* | Suicide vector to delete *tse1-tsi1* (*PA1844-5*) from *P. aeruginosa*, Sm^R^ | This study |
| pKNG101: *tse1-HA* | Suicide vector to add C-terminal *HA*-tag to *tse1* (*PA1844*) in *P. aeruginosa*, Sm^R^ | This study |
| pKNG101: *tse1-bla_TEM-1_* | Suicide vector to add C-terminal *bla_TEM-1_* to *tse1* (*PA1844*) in *P. aeruginosa*, Sm^R^ | This study |
| pKNG101: *tse1-HA-bla_TEM-1_* | Suicide vector to add C-terminal *HA*-tag and *bla_TEM-1_* to *tse1* (*PA1844*) in *P. aeruginosa*, Sm^R^ | This study |
| pKNG101: *tse1-HA-mScarlet-I* | Suicide vector to add C-terminal *HA*-tag and *mScarlet-I* to *tse1* (*PA1844*) in *P. aeruginosa*, Sm^R^ | This study |
| pKNG101: *hcp1^S115Q^* | Suicide vector to add serine to glutamine point mutation to residue 115 of *hcp1* (*PA0085*) in *P. aeruginosa*, Sm^R^ | This study |
| pKNG101: *tssB1-sfGFP* | Suicide vector to add C-terminal *sfGFP* to *tssB1* (*PA0083*) in *P. aeruginosa*, Sm^R^ | Laboratory collection |
| **Expression vectors** | | |
| pMMB67HE | Broad host range expression, P_LAC_ promoter, RSF1010 ori, Cb^R^ | (3) |
| pMMB67HE: *HA-tse4* | Expression strain for *tse4* with N-terminal *HA*-tag, Cb^R^ | This study |
| pMMB67HE: *tse4-HA* | Expression strain for *tse4* with C-terminal *HA*-tag, Cb^R^ | This study |
| pMMB67HE: *HA- CT1* | Expression strain for truncated *tse4* with N-terminal *HA*-tag, residues 171-195 deleted, Cb^R^ | This study |
| pMMB67HE: *HA-CT2* | Expression strain for truncated *tse4* with N-terminal *HA*-tag, residues 159-195 deleted, Cb^R^ | This study |
| pMMB67HE: *HA-CT3* | Expression strain for truncated *tse4* with N-terminal *HA*-tag, residues 139-195 deleted, Cb^R^ | This study |
| pMMB67HE: *HA-NT1* | Expression strain for truncated *tse4* with N-terminal *HA*-tag, residues 1-14 deleted, Cb^R^ | This study |
| pMMB67HE: *HA-NT2* | Expression strain for truncated *tse4* with N-terminal *HA*-tag, residues 1-37 deleted, Cb^R^ | This study |
| pME6032 | Broad host range expression, P_TAC_ promoter, P15A ori, Tc^R^ | (4) |
| pME6032: *hcp1* | Expression strain for *hcp1* for pull down control, Tc^R^ | This study |
| pME6032: *hcp1-FLAG* | Expression strain for *hcp1* with C-terminal *FLAG*-tag, Tc^R^ | This study |
| pME6032: *vgrG1b-FLAG* | Expression strain for *vgrG1b* with C-terminal *FLAG*-tag, Tc^R^ | This study |
| pME6032: *hcp2* | Expression strain for *hcp2* for pull down control, Tc^R^ | This study |
| pME6032: *hcp2-FLAG* | Expression strain for *hcp2* with C-terminal *FLAG*-tag, Tc^R^ | This study |
| pME6032: *vgrG2b-FLAG* | Expression strain for *vgrG2b* with C-terminal *FLAG*-tag, Tc^R^ | This study |
| pME6032: *hcp3* | Expression strain for *hcp3* for pull down control, Tc^R^ | This study |
| pME6032: *hcp3-FLAG* | Expression strain for *hcp3* with C-terminal *FLAG*-tag, Tc^R^ | This study |
| pME6032: *vgrG3-FLAG* | Expression strain for *vgrG3* with C-terminal *FLAG*-tag, Tc^R^ | This study |
| pACYCDuet^TM^-1 | High expression vector, with two multiple cloning sites (MCS), MCS-1 used here, T7 promoter, P15A Ori, Cm^R^ | Novagen |
| pACYC: *hcp1-his* | Expression strain for *hcp1* with C-terminal *his*-tag in pACYC-duet MCS1, Cm^R^ | This study |
| pACYC: *hcp1^S31Q^-his* | Expression strain for *hcp1^S31Q^* with C-terminal *his*-tag in pACYC-duet MCS1, Cm^R^ | This study |
| pACYC: *hcp1^T59Q^-his* | Expression strain for *hcp1^T59Q^* with C-terminal *his*-tag in pACYC-duet MCS1, Cm^R^ | This study |
| pACYC: *hcp1^S115Q^-his* | Expression strain for *hcp1^S115Q^* with C-terminal *his*-tag in pACYC-duet MCS1, Cm^R^ | This study |
| pACYC: *hcp2-his* | Expression strain for *hcp2* with C-terminal *his*-tag in pACYC-duet MCS1, Cm^R^ | This study |
| pACYC: *hcp2^T71Q^-his* | Expression strain for *hcp2^T71Q^* with C-terminal *his*-tag in pACYC-duet MCS1, Cm^R^ | This study |
| pACYC: *hcp3-his* | Expression strain for *hcp3* with C-terminal *his*-tag in pACYC-duet MCS1, Cm^R^ | This study |
| pACYC: *hcp3^T60Q^-his* | Expression strain for *hcp3^T60Q^* with C-terminal *his*-tag in pACYC-duet MCS1, Cm^R^ | This study |
| pET22b | High expression vector, T7 promoter, pBR322 ori, has N-terminal *pelB* leader sequence signal peptide, Ap^R^ | Novagen |
| pET22b: *tse1^C30A^-HA* | Expression strain for catalytic mutant (cysteine to alanine substitution at residue 30) of *tse1* with C-terminal *HA*-tag, *pelB* leader seq and *his*-tag removed from plasmid, Ap^R^ | This study |
| pET22b: *tse1^C30A^-HA-bla_TEM-1_* | Expression strain for catalytic mutant (cysteine to alanine substitution at residue 30) of *tse1* with C-terminal *HA*-tag and *bla_TEM-1_*, *pelB* leader seq and *his*-tag removed from plasmid, Ap^R^ | This study |
| pET22b: *HA-tse2^T79A S80A^* | Expression strain for catalytic mutant (threonine to alanine substitution at residue 79 and serine to alanine substitution at residue 80) of *tse2* with N-terminal HA-tag, *pelB* leader seq and *his*-tag removed from plasmid, Ap^R^ | This study |
| pET22b: *tse3^E250Q^-HA* | Expression strain for catalytic mutant (glutamic acid to glutamine substitution at residue 250) of *tse3* with C-terminal HA-tag, *pelB* leader seq and *his*-tag removed from plasmid, Ap^R^ | This study |
| pET22b: *HA-tse4* | Expression strain for *tse4* with N-terminal HA-tag, *pelB* leader seq and *his*-tag removed from plasmid, Ap^R^ | This study |
| pET22b: *tse4-HA* | Expression strain for *tse4* with C-terminal HA-tag, *pelB* leader seq and *his*-tag removed from plasmid, Ap^R^ | This study |
| pET22b: *HA-PA0256* | Expression strain for *PA0256* with N-terminal HA-tag, *pelB* leader seq and *his*-tag removed from plasmid, Ap^R^ | This study |
| pET22b: *PA0256-HA* | Expression strain for *PA0256* with C-terminal HA-tag, *pelB* leader seq and *his*-tag removed from plasmid, Ap^R^ | This study |
| pET22b: *HA-PA2066* | Expression strain for *PA2066* with N-terminal HA-tag, *pelB* leader seq and *his*-tag removed from plasmid, Ap^R^ | This study |
| pET22b: *PA2066-HA* | Expression strain for *PA2066* with C-terminal HA-tag, *pelB* leader seq and *his*-tag removed from plasmid, Ap^R^ | This study |
| pET22b: *HA-PA3440* | Expression strain for *PA3440* with N-terminal HA-tag, *pelB* leader seq and *his*-tag removed from plasmid, Ap^R^ | This study |
| pET22b: *PA3440-HA* | Expression strain for *PA3440* with C-terminal HA-tag, *pelB* leader seq and *his*-tag removed from plasmid, Ap^R^ | This study |
| pRL662: *GFP* | Broad host range vector producing GFP under a constitutive P_LAC_ promoter, pBBR1 ori, Gm^R^ | Laboratory collection |

1. Figurski DH, Helinski DR. 1979. Replication of an origin-containing derivative of plasmid RK2 dependent on a plasmid function provided in trans. Proc Natl Acad Sci U S A 76:1648-52.

2. Kaniga K, Delor I, Cornelis GR. 1991. A wide-host-range suicide vector for improving reverse genetics in gram-negative bacteria: inactivation of the blaA gene of Yersinia enterocolitica. Gene 109:137-41.

3. Furste JP, Pansegrau W, Frank R, Blocker H, Scholz P, Bagdasarian M, Lanka E. 1986. Molecular cloning of the plasmid RP4 primase region in a multi-host-range tacP expression vector. Gene 48:119-31.

4. Heeb S, Blumer C, Haas D. 2002. Regulatory RNA as mediator in GacA/RsmA-dependent global control of exoproduct formation in Pseudomonas fluorescens CHA0. J Bacteriol 184:1046-56.
